# Coordinating Mesopore Geometry and Interfacial Water Dynamics for Iron‐Site Evolution in Nitrate Electroreduction

**DOI:** 10.1002/anie.5865467

**Published:** 2026-09-19

**Authors:** Xiangyang Liu, Yingji Zhao, Norman C.‐R. Chen, Kevin C.‐W. Wu, Ho Ngoc Nam, Quan Manh Phung, Asep Sugih Nugraha, Yusuke Asakura, Yusuke Yamauchi

**Affiliations:** ^1^ Department of Materials Process Engineering, Graduate School of Engineering Nagoya University Nagoya Japan; ^2^ Molecular Science and Technology Program Taiwan International Graduate Program Academia Sinica Taipei Taiwan; ^3^ International Graduate Program of Molecular Science and Technology (NTU‐MST) National Taiwan University Taipei Taiwan; ^4^ Department of Chemical Engineering National Taiwan University Taipei Taiwan; ^5^ Center of Atomic Initiative for New Materials (AI‐MAT) National Taiwan University Taipei Taiwan; ^6^ Department of Chemistry Graduate School of Science Nagoya University Nagoya Japan; ^7^ Institute of Transformative Bio‐Molecules (WPI‐ITbM) Nagoya University Nagoya Japan; ^8^ Australian Institute for Bioengineering and Nanotechnology (AIBN) The University of Queensland Brisbane Queensland Australia; ^9^ Department of Convergent Biotechnology and Advanced Materials Science Kyung Hee University Yongin‐si Gyeonggi‐do South Korea

**Keywords:** dynamic active sites, electrocatalytic nitrate reduction, interfacial water, mesoporous materials, metal–organic frameworks

## Abstract

Developing electrocatalysts with active sites that undergo structural evolution under operating conditions remains a challenge in electrochemical nitrate reduction. Here, we use single‐crystalline mesoporous metal–organic frameworks (MOFs) with distinct pore geometries to investigate the potential‐dependent evolution of iron active sites during electrochemical nitrate‐to‐ammonia conversion. Fe single atoms are anchored within hexagonal and cubic mesoporous frameworks, providing two structurally distinct environments for examining their catalytic and working‐state responses. *Operando* FTIR, H/D isotope‐exchange, and DRT analyses reveal that the 1D hexagonal mesoporous environment is associated with a distinct interfacial‐water response and proton‐involved reaction behavior. This response coincides with changes in the average Fe coordination environment. *Operando* XAS further reveals potential‐dependent changes in both catalysts, with FeSAs@*m*UiO‐P exhibiting a more pronounced Fe‐associated higher‐shell contribution and greater post‐potential recovery. Under the investigated conditions, FeSAs@*m*UiO‐P achieves a Faradaic efficiency of 99.1% at −1.05 V versus RHE and exhibits higher NH_3_ selectivity than its cubic counterpart. Collectively, these findings reveal a potential‐dependent correspondence among mesopore architecture, interfacial water organization, and Fe‐site evolution. Although the causal relationship between water organization and Fe‐site evolution remains to be established, this correspondence provides a working framework for investigating reaction‐state‐dependent catalytic interfaces.

## Graphical abstract

Mesopore geometry programs the working‐state interface of Fe single sites during nitrate electroreduction. Hexagonal channels reorganize interfacial water and accompany the reversible evolution of isolated Fe─O_4_‐like sites toward Fe─O─Fe‐like motifs, promoting successive hydrogenation toward NH_3_ with a Faradaic efficiency of 99.1%.

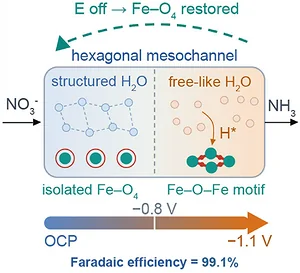

## 1 Introduction

Electrochemical nitrate reduction reaction (NitRR) offers a sustainable pathway for ambient ammonia synthesis, serving as a promising alternative to the carbon‐intensive Haber–Bosch process [1, 2, 3, 4, 5]. However, developing electrocatalysts that reconcile the trade‐off between activity and selectivity remains a formidable bottleneck [6, 7, 8]. Single‐atom catalysts (SACs), while maximizing atomic utilization and suppressing the competing hydrogen evolution reaction (HER), often suffer from sluggish hydrogenation kinetics due to their discrete active centers [9, 10, 11]. Conversely, multinuclear metal configurations possess continuous electronic states that facilitate multi‐electron transfer but typically lack the selectivity required for efficient ammonium production [12, 13, 14]. Bridging the gap between the high selectivity of isolated atoms and the high activity of multinuclear metal configurations is thus essential for advancing electrosynthesis technologies.

A promising strategy to overcome this limitation is to engineer dynamic active sites that undergo reversible structural reconstruction under operating conditions [15, 16, 17]. Conceptually, if isolated atoms can transiently aggregate into multinuclear active motifs to accelerate rate‐limiting steps and subsequently re‐disperse, the catalyst could ideally adapt to reaction demands. However, controlling such dynamic evolution to prevent irreversible agglomeration poses a significant challenge. The evolution of dynamic active sites can be influenced by their local reaction environment. In proton‐intensive reactions like the nitrate reduction reaction, water acts as a field‐responsive mediator that regulates proton availability and dictates the kinetics of proton‐coupled electron transfer. Consequently, the stability and population of transient Fe‐associated environments may depend in part on the local hydrogen‐bonding and intermediate‐coverage conditions. Any mismatch in proton supply can lead to intermediate accumulation, ultimately destabilizing or suppressing these reconstructed active sites.

To isolate and interrogate this microenvironmental regulation, a structurally ordered platform that enables comparative analysis of architecture‐dependent interfacial responses is needed. Single‐crystalline mesoporous metal–organic frameworks (MOFs) provide such a platform with unusual precision. Unlike microporous materials that sterically hinder active‐site reconstruction, mesopores (2–50 nm) provide accessible nanospaces for dynamic site reconstruction and mass transport [18, 19, 20, 21, 22, 23]. Crucially, the grain‐boundary‐free nature of single crystals ensures continuous, long‐range ordered diffusion pathways, allowing us to comparatively examine the responses of hexagonal versus cubic confinement on interfacial dynamics with reduced grain‐boundary‐associated heterogeneity [24, 25, 26, 27]. This structural precision provides a unique opportunity to directly probe how mesopore‐enabled confinement orchestrates the synergy between proton supply and active‐site reconstruction.

In this study, we use Fe single atoms supported within hexagonal and cubic mesoporous UiO frameworks to examine the potential‐dependent correspondence among pore architecture, interfacial water, and Fe coordination during NitRR. We synthesize Fe single atoms anchored within hexagonal and cubic mesopores (Figure 1a). The Na^+^ species shown in Figure 1a schematically represent hydrated electrolyte ions confined within the mesoporous channels and are included to illustrate the interfacial solvation environment surrounding the Fe active sites. *Operando* spectroscopy, H/D isotope‐exchange measurements, and DRT studies reveal that the 1D hexagonal channels are associated with a distinct proton‐involved interfacial response through the regulation of interfacial water organization. These differences in interfacial response coincide with distinct potential‐dependent changes in the average Fe coordination environments, with the hexagonal catalyst exhibiting a more pronounced Fe‐associated higher‐shell contribution and greater post‐potential recovery than its cubic counterpart. Consequently, the hexagonal catalyst achieves a Faradaic efficiency (FE) of 99.1% and a superior ammonia yield. Our work identifies a possible connection linking mesopore geometry, interfacial water dynamics, and active‐site adaptability, offering a design consideration for potential‐responsive electrocatalytic interfaces.

**FIGURE 1:**
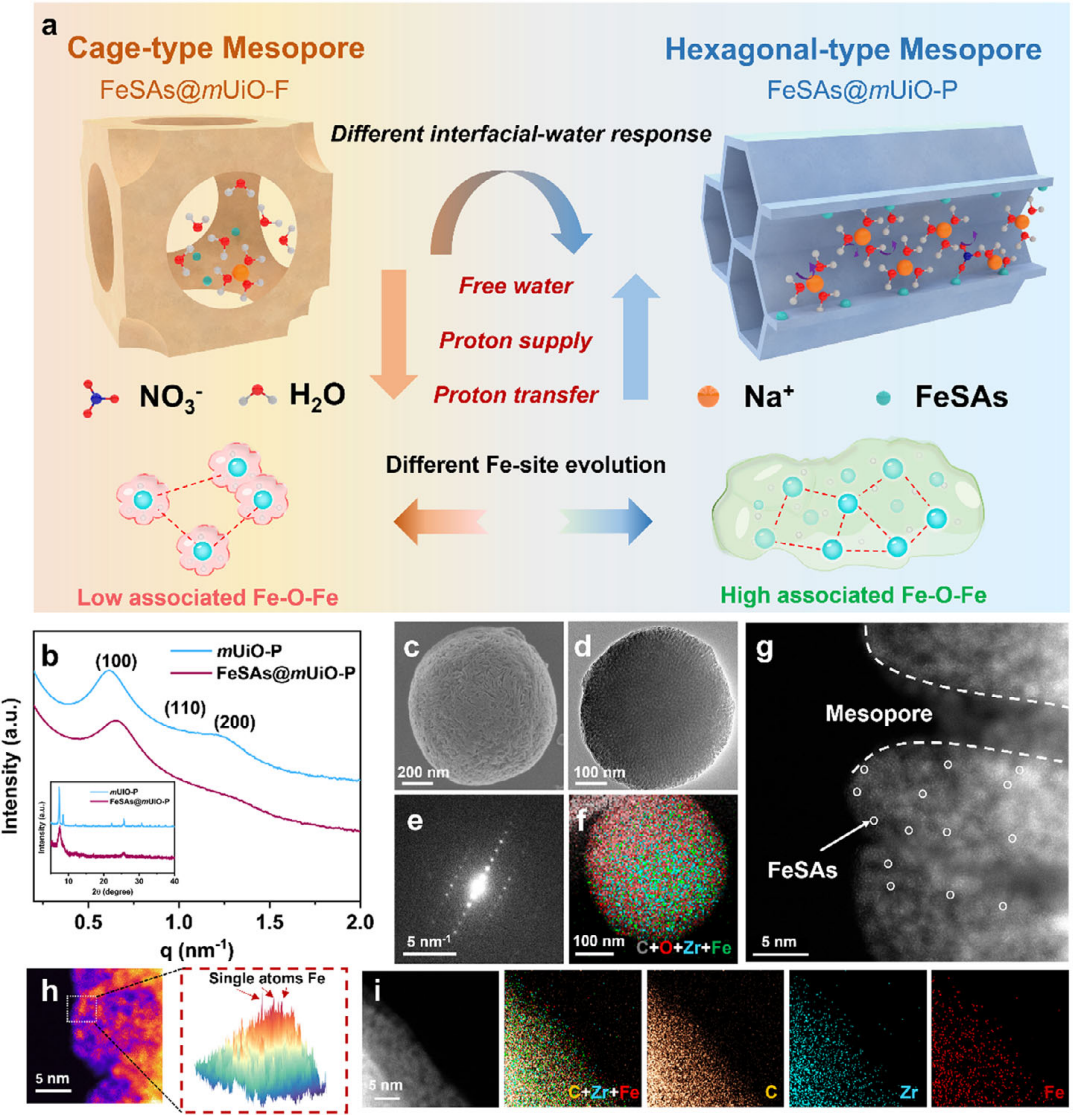
Structural design of single‐crystalline mesoporous MOFs with symmetry‐distinct geometries. (a) Schematic illustration of cage‐type and hexagonal‐type mesoporous frameworks. (b) SAXS patterns and (inset) XRD patterns of FeSAs@*m*UiO‐P and *m*UiO‐P. (c) SEM; (d) TEM; (e) SAED; (f) EDS; and (g) HR‐STEM images of FeSAs@*m*UiO‐P. (h) 3D visualization of dispersion of single Fe atoms. (i) HR‐EDS of FeSAs@*m*UiO‐P and *m*UiO‐P.

## 2 Results and Discussion

### 2.1 Symmetry‐Controlled Synthesis of Single‐Crystalline Mesoporous MOFs

Single‐crystalline mesoporous frameworks, *m*UiO‐P and *m*UiO‐F, were synthesized via a hydrothermal approach using P‐105 and F‐127 block copolymers as structure‐directing agents to induce hexagonal and cubic symmetries, respectively. Subsequently, Fe species were introduced into the as‐synthesized MOF particles, yielding FeSAs@*m*UiO‐P and FeSAs@*m*UiO‐F. The small‐angle x‐ray scattering (SAXS) patterns of the materials before and after Fe loading are shown in Figures 1b and S1. For FeSAs@*m*UiO‐P, three well‐defined scattering peaks were observed at *q* = 0.652, 1.058, and 1.326 nm^−1^, corresponding to the (100), (110), and (200) reflections of the 2D hexagonal mesostructure [28]. In contrast, FeSAs@*m*UiO‐F displayed characteristic scattering peaks at *q* = 0.572, 0.863, and 0.992 nm^−1^, which could be indexed a typical 3D cubic mesostructure. Importantly, the SAXS patterns of *m*UiO‐P and *m*UiO‐F closely resembled those of their Fe‐loaded counterparts, indicating that the average mesostructural periodicity was largely retained after Fe incorporation. While XRD patterns (Figures 1b and S2) matched the crystalline UiO‐66‐Zr framework [29], the observed peak broadening after Fe incorporation suggests local lattice distortion and microstrain arising from the altered charge distribution and steric hindrance around Fe‐coordinated Zr nodes. Importantly, no diffraction peaks attributable to crystalline Fe‐containing phases were detected by XRD.

Morphological characterization by scanning electron microscopy (SEM) (Figures 1c and S3) revealed that both catalysts consisted of ∼400 nm spherical particles with ordered mesoporous architectures. Obvious mesopores were observed on the particle surfaces, albeit with distinct structural features. FeSAs@*m*UiO‐P exhibited an ordered array of uniformly distributed 1D channels, whereas FeSAs@*m*UiO‐F displayed well‐defined circular mesopores. Low‐magnification SEM images (Figure S4) reveal that the FeSAs@*m*UiO‐P and FeSAs@*m*UiO‐F catalysts consist of uniform spherical particles, indicating excellent morphological uniformity and dispersion. Transmission electron microscopy (TEM) was further employed to confirm these structural differences (Figure 1d; Figures S5 and S6). The mesoporous channels of FeSAs@*m*UiO‐P appeared to intersect in the top‐view projection, although they were not physically connected. In contrast, FeSAs@*m*UiO‐F exhibited isolated straight channels extending toward the particle core. The single‐crystalline nature of the FeSAs@*m*UiO‐P particles is evidenced by the sharp, ordered array of diffraction spots in the SAED pattern (Figure 1e). This well‐defined diffraction lattice, along with the absence of diffuse halos or ring‐like features, confirms a high degree of long‐range order free from amorphous domains or grain boundaries. Such a single‐crystalline structure ensured uniform pore alignment and identical crystallographic orientation throughout each particle, leading to well‐defined and continuous diffusion channels for electrolyte ions and reactants. Energy‐dispersive x‐ray spectroscopy (EDS) mapping revealed that both samples possessed a uniform distribution of all constituent elements, showing a spatially homogeneous Fe distribution at the probed length scale (Figures 1f and S7). Atomic‐resolution scanning transmission electron microscopy (STEM) (Figure 1g) further verified that Fe atoms are homogeneously distributed as isolated single sites coordinated to the Zr─O nodes of the framework. Three‐dimensional visualization (Figure 1h) and high‐resolution energy‐dispersive spectroscopy (HR‐EDS) mapping (Figure 1i) of FeSAs@*m*UiO‐P further supported the spatially homogeneous distribution of Fe within the mesoporous host at the probed length scale.

### 2.2 Atomic Identification of Isolated Fe─O_4_‐Like Active Centers

X‐ray photoelectron spectroscopy (XPS) was conducted to investigate the elemental composition and bonding states. For both catalysts, the Fe 2p spectrum could be deconvoluted into four distinct peaks, corresponding to the 2p_3/2_, 2p_3/2_ satellite, 2p_1/2_, and 2p_1/2_ satellite orbitals in the range of 705–740 eV (Figure S8) [30]. The Fe contents calculated from XPS were 2.0 wt% for FeSAs@*m*UiO‐P and 1.5 wt% for FeSAs@*m*UiO‐F (Table S1). These values were consistent with the elemental proportions determined by inductively coupled plasma optical emission spectroscopy (ICP‐OES), which were 1.5 and 1.2 wt%, respectively (Table S2). XPS surveys of both samples also included C 1s, O 1s, and F 1s regions, and the two catalysts showed nearly identical surface compositions, indicating that Fe loading did not change the MOF surface chemistry (Figures S9 and S10). The N_2_ adsorption–desorption isotherms and corresponding pore size distributions (Figure S11, Table S3) indicated that both *m*UiO‐P and *m*UiO‐F exhibited typical type‐IV curves, confirming their mesoporous nature [31]. After Fe incorporation, the overall shapes of the isotherms and the pore size distributions remained almost unchanged, suggesting that the mesostructures were well preserved. The dominant peaks in the pore‐size‐distribution profiles remained at similar positions after Fe incorporation, indicating that the characteristic mesopore dimensions were largely retained. Nevertheless, the quantitative pore‐volume contributions differed appreciably between the two Fe‐loaded catalysts. FeSAs@*m*UiO‐P exhibited a mesopore volume of 0.3127 cm^3^ g^−1^, nearly twice that of FeSAs@*m*UiO‐F (0.1620 cm^3^ g^−1^). Mesopores accounted for approximately 76.0% of the total pore volume in FeSAs@*m*UiO‐P, compared with 52.8% in FeSAs@*m*UiO‐F, indicating that the accessible pore structure of the P‐type catalyst was more strongly dominated by the mesoporous contribution. This larger absolute and relative mesopore volume may facilitate electrolyte penetration, nitrate transport, and access to Fe sites within the porous framework. Therefore, this textural difference may also contribute to the measured catalytic performance, and its contribution cannot be quantitatively separated from that of mesopore architecture in the present comparison.

To probe the local coordination environment and interfacial structure, x‐ray absorption spectroscopy (XAS) measurements were conducted. The normalized Fe K‐edge x‐ray absorption near‐edge structure (XANES) spectra are shown in Figure 2a, together with the reference samples of Fe_2_O_3_, FeO, and Fe foil. Both catalysts exhibited an absorption edge position and spectral shape most similar to that of Fe_2_O_3_, indicating that Fe centers predominantly existed in the +3 oxidation state. Moreover, a pre‐edge feature located at ∼7117 eV could be assigned to the 1s → 3d transition, characteristic of Fe^3+^ species in a non‐centrosymmetric oxygen coordination environment (3d^5^ configuration) [32]. *K* ^2^‐weighted extended x‐ray absorption fine structure (EXAFS) oscillation functions (χ(k)) of FeSAs@*m*UiO‐P, FeSAs@*m*UiO‐F, and reference compounds are shown in Figure S12. Both samples exhibited similar oscillation profiles distinct from those of Fe foil, indicating the absence of Fe─Fe coordination and confirming the atomically dispersed nature of Fe sites. The oscillation phase and amplitude were closer to Fe_2_O_3_, suggesting that Fe atoms were mainly coordinated with oxygen in an Fe─O environment. The Fourier‐transformed EXAFS spectra were further employed to analyze the coordination structure (Figure 2b). Both samples displayed a prominent peak at ∼1.8 Å, which was ascribed to the first‐shell Fe─O_4_‐like coordination in Fe^3+^. Importantly, no discernible peak corresponding to the Fe─Fe scattering observed for metallic Fe foil at approximately 2.4 Å was detected, indicating the absence of detectable Fe aggregation within the sensitivity of the measurement.

**FIGURE 2:**
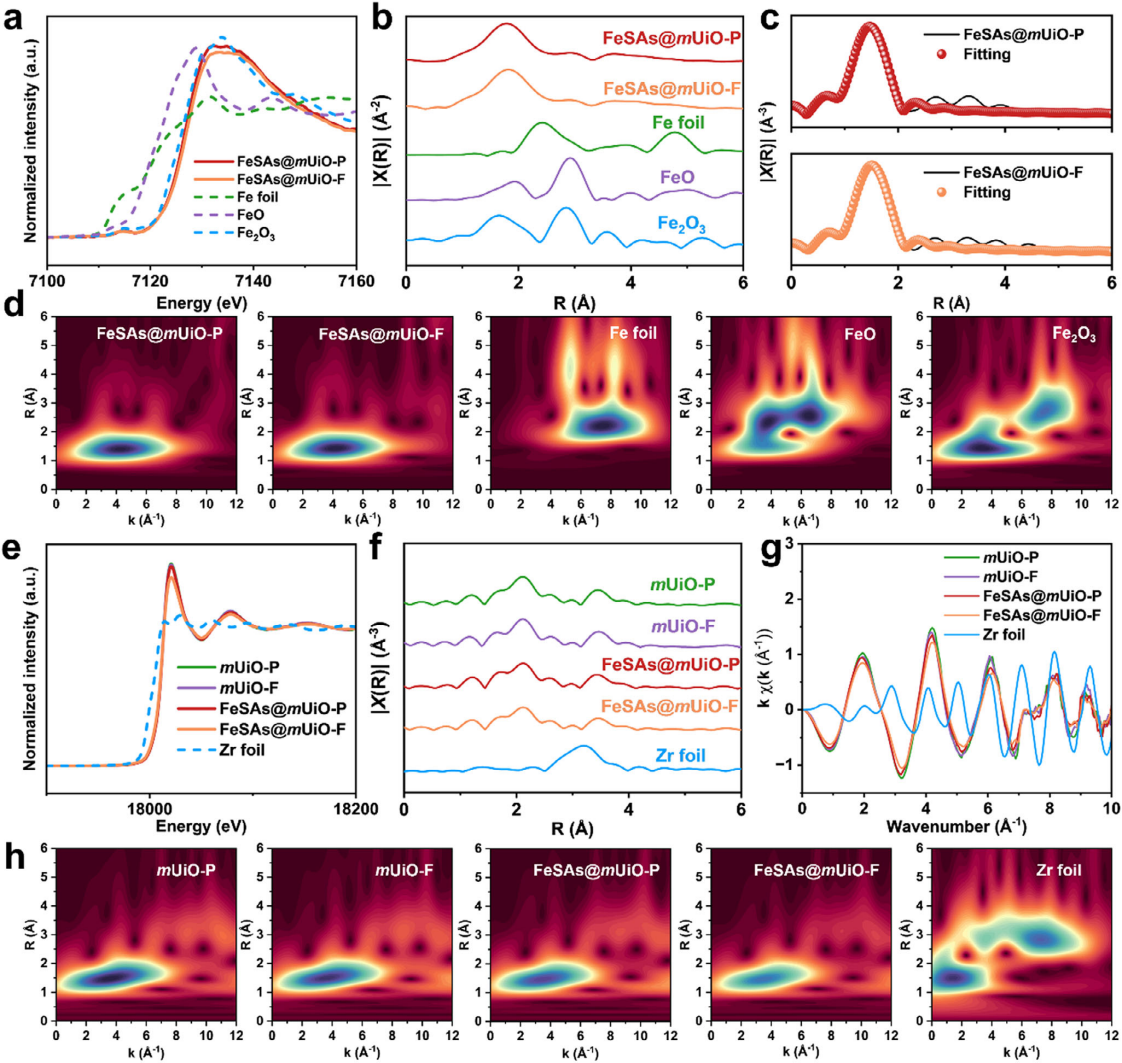
Identification of atomically dispersed Fe─O_4_ active sites within mesoporous frameworks. (a) XANES spectra at the Fe K‐edge, (b) FT‐EXAFS spectra at the Fe K‐edge, (c) EXAFS fittings and (d) Wavelet‐transform (WT) maps of Fe K‐edge of FeSAs@*m*UiO‐P, FeSAs@*m*UiO‐F with reference samples (Fe foil, FeO, Fe_2_O_3_). (e) XANES spectra at the Zr K‐edge, (f) FT‐EXAFS spectra at the Zr K‐edge, (g) *k* ^2^‐weighted EXAFS oscillation curves at the Zr K‐edge and (h) Wavelet‐transform (WT) maps of Zr K‐edge of *m*UiO‐P, *m*UiO‐F, FeSAs@*m*UiO‐P, FeSAs@*m*UiO‐F with reference samples (Zr foil).

The FT‐EXAFS fitting results are presented in Figure 2c and Table S4. Owing to the EXAFS phase shift, the radial distances displayed in the Fourier‐transformed spectra are generally about 0.3 Å smaller than the corresponding crystallographic distances. For both samples, the best‐fit models yielded a coordination number of 4.5, consistent with an Fe─O_4_‐like coordination structure. The corresponding Fe─O bond lengths were determined to be 2.00 Å for FeSAs@*m*UiO‐P and 2.02 Å for FeSAs@*m*UiO‐F. The Fe K‐edge wavelet transform (WT) of the EXAFS data provided a 2D representation in both k and R spaces (Figure 2d). WT of FeSAs@*m*UiOs displayed a strong intensity maximum in the low‐k region at R ≈ 1.5 Å, which could be assigned to Fe─O coordination similar to Fe_2_O_3_, while no discernible high‐k feature at R ≈ 2.2 Å was observed. This confirmed that Fe atoms were atomically dispersed without detectable Fe─Fe contributions. A similar analysis was performed for the Zr K‐edge. As shown in Figure 2e, the spectra of both *m*UiOs and FeSAs@*m*UiOs samples differed markedly from that of Zr foil, confirming that Zr atoms were predominantly coordinated with oxygen rather than forming metallic Zr─Zr bonds. This observation was consistent with the FT‐EXAFS results, where no peak corresponding to metallic Zr─Zr scattering at ∼3.2 Å was detected (Figure 2f). In contrast, a higher‐shell contribution corresponding to a fitted Zr─Zr distance of approximately 3.5 Å was observed, which was assigned to the intrinsic framework Zr─Zr correlation within the UiO‐type Zr_6_ nodes. Quantitative Zr K‐edge EXAFS fitting incorporating the framework Zr─Zr scattering path yielded a coordination number of 4 for all samples (Figure S13, Table S5). The fitted Zr─Zr distances were approximately 3.5 Å for all samples. These values are consistent with the fourfold framework Zr─Zr correlation expected for UiO‐type Zr_6_ nodes and indicate that their basic connectivity was retained after Fe incorporation. Furthermore, the χ(k) oscillations of the samples exhibited a different frequency compared with Zr foil, reflecting distinct backscattering paths (Figure 2g). Notably, after Fe loading, the overall WT intensity of Zr decreased (Figure 2h). This phenomenon could be attributed to a perturbation of the average Zr coordination environment, which may involve increased structural disorder.

### 2.3 Mesopore Geometry‐Dependent NitRR Efficiency

The electrocatalytic performance of FeSAs@*m*UiO‐P and FeSAs@*m*UiO‐F was evaluated in 0.5 M Na_2_SO_4_ with and without 0.1 M NaNO_3_. The concentrations of NO_3_^−^, NO_2_^−^, and NH_3_ were quantified based on their respective calibration curves obtained from standard solutions, ensuring the accuracy and reliability of the measured results (Figures S14–S16). To evaluate the catalytic performance of FeSAs@*m*UiO‐P and FeSAs@*m*UiO‐F, linear sweep voltammetry (LSV) was conducted. In bare Na_2_SO_4_ solutions only HER proceeded, as shown by the dashed lines in Figure 3a. The current density and onset potential of FeSAs@*m*UiO‐F were slightly higher than that of FeSAs@*m*UiO‐P, indicating the higher HER activity of FeSAs@*m*UiO‐F. However, after the addition of 0.1 M NaNO_3_ electrolyte, NitRR occurred simultaneously with HER with a competing relationship. The relative current‐density and onset‐potential trends of the two catalysts were reversed after nitrate addition, suggesting that their distinct pore structures may contribute to different interfacial responses under HER and NitRR conditions. Because nitrate reduction involves a multistep proton‐coupled pathway, it may be particularly sensitive to the local transport and interfacial conditions within the pores. The confined 1D channels of FeSAs@*m*UiO‐P may contribute to its higher NH_3_ Faradaic efficiencies and yield rates over the investigated potential range. FeSAs@*m*UiO‐P exhibited a higher FE and NH_3_ yield rate than FeSAs@*m*UiO‐F across the entire potential range (Figures 3b,c and S17). At less negative potentials, both catalysts showed modest FE values, indicating that the potential was insufficient to drive the multistep proton‐coupled reduction for the NitRR, thereby leading to incomplete reduction of nitrate and accumulation of nitrite. A pronounced increase in FE was observed between −0.8 and −0.9 V versus RHE for both catalysts. FeSAs@*m*UiO‐P achieved a FE of 99.1% at −1.05 V versus RHE, whereas FeSAs@*m*UiO‐F reached only 85%. At potentials more negative than −1.1 V versus RHE, both catalysts exhibited decreased NH_3_ Faradaic efficiencies. This decrease was accompanied by a greater contribution from HER, indicating increased competition between nitrate reduction and H_2_ formation at more negative potentials. In both cases, the NH_3_ yield rate increased with more negative potentials, but FeSAs@*m*UiO‐P (182 µmol h^−1^ cm^−2^ at −1.05 V vs. RHE) outperformed FeSAs@*m*UiO‐F (151 µmol h^−1^ cm^−2^ at −1.05 V vs. RHE), consistent with a greater preference for nitrate reduction over the competing HER under these conditions. To further distinguish the contributions of nitrate reduction and hydrogen evolution, the corresponding partial current densities were calculated (Figure S18). FeSAs@*m*UiO‐P exhibited consistently higher NH_3_ partial current densities than FeSAs@*m*UiO‐F across the investigated potential range, whereas higher H_2_ partial current densities were generally observed for FeSAs@*m*UiO‐F, particularly at more negative potentials. These results further indicated that the hexagonal mesoporous architecture is consistent with a greater preference for nitrate reduction over the competing HER process. To further assess the balance between NH_3_ selectivity and electrical energy input, the estimated half‐cell energy efficiencies were calculated at different potentials (Table S6). FeSAs@*m*UiO‐P exhibited higher values than FeSAs@*m*UiO‐F over the investigated potential range and reached a maximum of approximately 23.9% at −1.00 V versus RHE. The subsequent decrease at more negative potentials indicated that the increased electrical energy input was not fully compensated by the corresponding NH_3_ FE. To evaluate the contribution of the mesoporous framework itself, Fe‐free *m*UiO‐P and *m*UiO‐F were examined as control samples (Figure S19). A similar performance trend (*m*UiO‐P > *m*UiO‐F) was observed, suggesting that the mesoporous architecture influenced the nitrate reduction process. Nevertheless, the marked enhancement in catalytic activity after Fe incorporation supports the Fe species as the predominant active centers, while the Fe‐free control results indicate that the mesoporous architecture may also modulate the local reaction environment.

**FIGURE 3:**
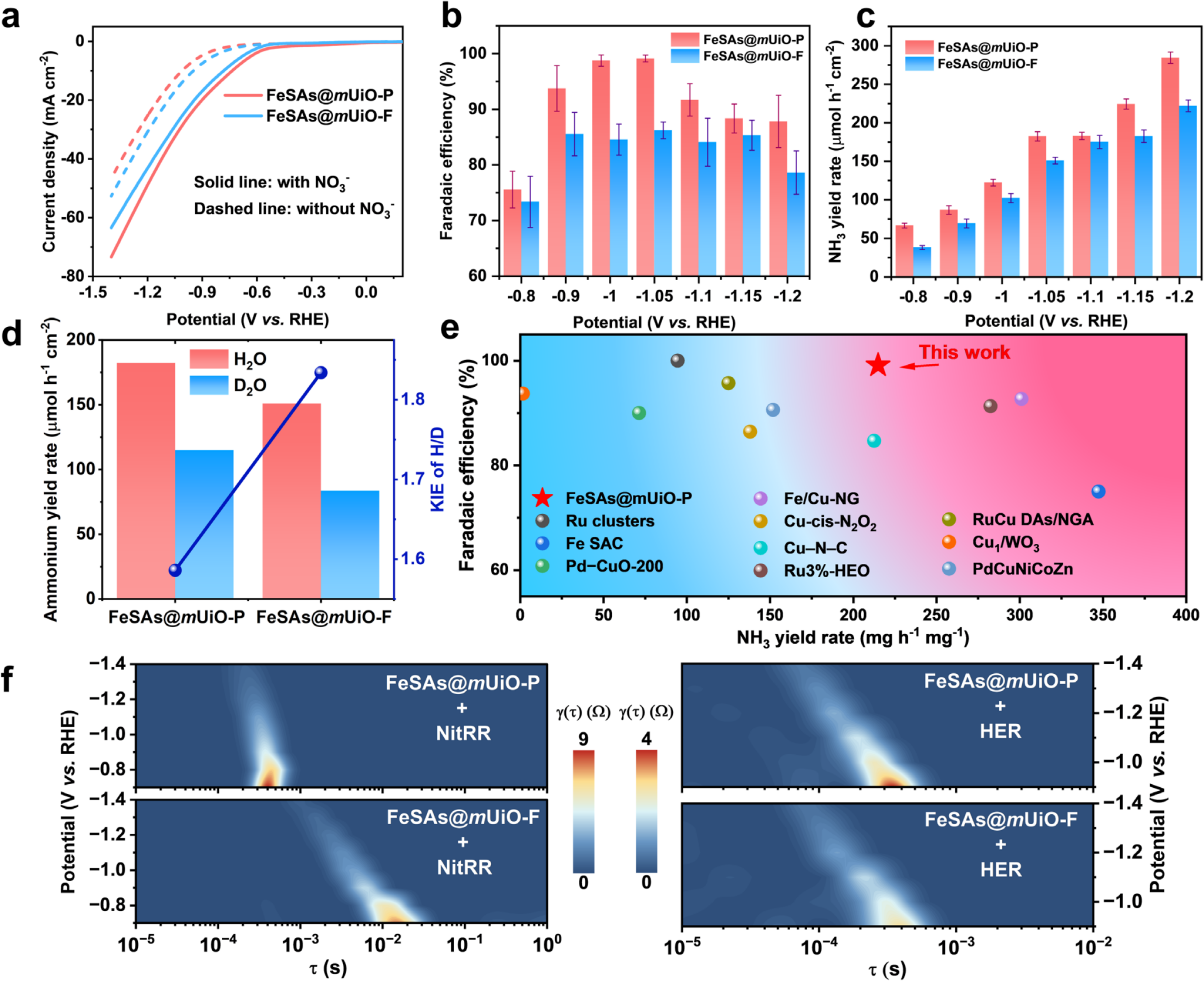
Mesopore geometry‐dependent nitrate reduction performance and interfacial kinetics. (a) LSV curves of FeSAs@*m*UiO‐P and FeSAs@*m*UiO‐F in 0.5 M Na_2_SO_4_ electrolyte with and without the addition of 0.1 M NaNO_3_. (b) FE and (c) ammonium yield rate of FeSAs@*m*UiO‐P and FeSAs@*m*UiO‐F under different potentials. (d) KIE of H/D and corresponding NH_3_ yield rates of FeSAs@*m*UiO‐P and FeSAs@*m*UiO‐F under −1.05 V versus RHE. (e) Comparison of FE and ammonium yield rate of FeSAs@*m*UiO‐P and other reported catalysts. (f) DRT analysis of FeSAs@*m*UiO‐P and FeSAs@*m*UiO‐F in NitRR and HER processes.

The influence of nitrate availability on the catalytic response was further examined at nitrate concentrations of 10, 100, and 500 mM. For both catalysts, increasing the nitrate concentration produced a larger cathodic current density, particularly in the more negative potential region (Figure S20). FeSAs@*m*UiO‐P generally exhibited a larger current response than FeSAs@*m*UiO‐F under otherwise identical conditions. Because the total current may also contain contributions from competing reactions, NH_3_‐selective measurements were performed at 500 mM nitrate. FeSAs@*m*UiO‐P maintained higher NH_3_ Faradaic efficiencies and yield rates than FeSAs@*m*UiO‐F over the investigated potential range (Figure S21). Compared with the results obtained at 100 mM, the higher nitrate concentration increased the NH_3_ yield rates but resulted in lower NH_3_ FEs. Such concentration‐dependent behavior is consistent with previous reports showing that increased nitrate availability can enhance the cathodic current and NH_3_ production rate, while excessively high nitrate concentrations may decrease the NH_3_ FE by limiting the complete conversion of accumulated nitrogen‐containing intermediates [33, 34]. To account for their different Fe loadings, the NH_3_ yield rates were further normalized to the total Fe content (Figure S22). At 100 mM nitrate, FeSAs@*m*UiO‐P and FeSAs@*m*UiO‐F exhibited relatively similar apparent Fe‐normalized turnover frequencies, suggesting that the activity difference was less distinguishable under this substrate concentration. When the nitrate concentration was increased to 500 mM, FeSAs@*m*UiO‐P exhibited consistently higher apparent TOFs than FeSAs@*m*UiO‐F over the investigated potential range. The more pronounced difference at 500 mM suggests that increased nitrate availability allowed the distinct catalytic responses of the two materials to become more evident. A similar trend was observed after normalization to the N_2_‐derived BET surface area (Figure S23). The two catalysts exhibited comparable BET‐area‐normalized NH_3_ yield rates at 100 mM nitrate, whereas FeSAs@*m*UiO‐P showed consistently higher values at 500 mM nitrate. Together, the Fe‐content‐normalized and BET‐area‐normalized results indicate that the performance difference under increased nitrate availability cannot be accounted for by either Fe loading or total BET surface area alone. To further examine the influence of Fe loading, FeSAs@*m*UiO‐F samples with different Fe contents were evaluated under otherwise identical conditions (Figure S24). The cathodic current density, NH_3_ FE, and NH_3_ yield rate did not increase monotonically with increasing Fe loading. Instead, the sample with an intermediate Fe loading exhibited the highest NH_3_ yield rates across the investigated potential range, whereas both lower and higher Fe loadings resulted in reduced performance. These results indicate that the total Fe content alone was insufficient to determine the nitrate reduction performance.

To gain deeper insight into the role of proton transfer during nitrate reduction, kinetic isotope effect (KIE) measurements were performed by replacing H_2_O with D_2_O (Figure 3d). A measurable decrease in catalytic activity was observed for both catalysts in D_2_O‐based electrolyte, supporting the kinetic relevance of proton‐involved steps in the reaction process. Notably, FeSAs@*m*UiO‐F exhibited a larger KIE value (1.83) than FeSAs@*m*UiO‐P (1.58), indicating a stronger kinetic dependence on proton transfer. The smaller KIE observed for FeSAs@*m*UiO‐P indicates a lower apparent sensitivity to H/D substitution, consistent with distinct proton‐involved kinetics in the hexagonal mesoporous environment.

To further identify the nitrogen source of the generated ammonia, isotope‐labeling experiments were conducted using Na^15^NO_3_ and Na^14^NO_3_ as the nitrogen precursors, followed by ^1^H NMR analysis. As shown in Figure S25, a typical triplet signal with a coupling constant of 51 Hz was observed when Na^14^NO_3_ was used, corresponding to the formation of ^14^NH_4_^+^. In contrast, when Na^15^NO_3_ was employed, the signal shifted to a characteristic doublet with a coupling constant of 72 Hz, which was consistent with ^15^NH_4_^+^. These results unequivocally confirmed that the detected NH_3_ originated from the electroreduction of nitrate ions, rather than from external nitrogen contaminants. In contrast, no characteristic absorption peaks were detected in the UV–vis spectrum of the electrolyte without NO_3_^−^ after electrolysis, further confirming that the generated NH_3_ originated exclusively from the electroreduction of nitrate rather than from other nitrogen sources or contaminants (Figure S26). The durability of FeSAs@*m*UiO‐P was evaluated over 10 consecutive cycles at −1.05 V versus RHE in 0.5 M Na_2_SO_4_ and 0.1 M NaNO_3_ electrolyte. As shown in Figure S27, the FE remained above 93% throughout all cycles, accompanied by nearly identical NH_3_ yields, indicating that the activity and selectivity were retained over the 10‐cycle test. A comparison with previously reported nitrate‐reduction electrocatalysts is provided in Figure 3e and Table S7 [13, 35, 36, 37, 38, 39, 40, 41, 42, 43]. In the present work, the mass‐normalized NH_3_ yield rate of 214.86 mg h^−1^ mg_Fe_^−1^ is internally consistent with the geometric‐area‐normalized value of 182 µmol h^−1^ cm^−2^, based on a catalyst loading of 0.96 mg_cat_ cm^−2^ and an Fe content of 1.5 wt%.

Electrochemical active surface area (ECSA) of the as‐prepared catalysts was evaluated by measuring the electrochemical double‐layer capacitance (*C*_dl_) via cyclic voltammetry (CV) in the non‐faradaic region (0.50–0.60 V vs. RHE) at various scan rates from 5 to 50 mV s^−1^ (Figures S28 and S29). As illustrated in the linear fitting of the capacitive currents (Δ*j*/2) against scan rates, the *C*_dl_ value of FeSAs@*m*UiO‐P was calculated to be 280.15 µF cm^−2^, which is higher than that of FeSAs@*m*UiO‐F (172.90 µF cm^−2^). For a direct comparison, the principal compositional, textural, electrochemical, and catalytic parameters of the two catalysts are summarized in Table S8. However, these measurements provide limited information on how the catalyst interface and proton‐coupled process changed under reaction conditions. Potential‐dependent electrochemical impedance spectroscopy (EIS) and distribution of relaxation times (DRT) analyses were therefore performed to further examine their interfacial responses under HER and NitRR conditions.

As shown in Figures S30 and S31, both catalysts exhibited depressed semicircular Nyquist responses over the investigated potential range in nitrate‐free and nitrate‐containing electrolytes, indicating nonideal and distributed interfacial behavior. The impedance spectra were fitted using an equivalent circuit consisting of *R*_1_, *R*_2_, and a constant‐phase element (CPE). In this phenomenological circuit, *R*_1_ approximates the series resistance arising from the electrolyte, electrode, and electrical contacts. *R*_2_ describes the interfacial polarization response, which may involve coupled charge‐transfer and transport processes, while the CPE accounts for nonideal interfacial capacitance. With increasing cathodic polarization, the Nyquist arcs of both catalysts generally contracted and the fitted *R*_2_ values decreased, consistent with a reduction in the overall interfacial polarization resistance. Notably, the relative *R*_2_ values of FeSAs@*m*UiO‐P and FeSAs@*m*UiO‐F depended on the reaction environment. At potentials more positive than −1.0 V versus RHE, FeSAs@*m*UiO‐F exhibited a smaller *R*_2_ under nitrate‐free HER conditions, whereas FeSAs@*m*UiO‐P showed a smaller *R*_2_ after nitrate addition. At −1.0 V and more negative potentials, the fitted *R*_2_ values of the two catalysts became comparable within each electrolyte. The reversal in relative *R*_2_ after nitrate addition demonstrates that the interfacial polarization response is reaction‐dependent rather than governed by a universal conductivity or resistance advantage. The convergence of *R*_2_ at more negative potentials further indicates that the difference in NH_3_ selectivity cannot be described by the total interfacial polarization resistance alone.

To further probe the interfacial kinetics and transport processes, Bode plots were constructed from the EIS data (Figures S32 and S33). In the presence of NO_3_^−^, both catalysts exhibited characteristic phase responses shifting toward higher frequencies with increasing cathodic polarization, consistent with an increasing contribution from nitrate‐associated interfacial processes. Notably, FeSAs@*m*UiO‐P displayed a higher‐frequency response than FeSAs@*m*UiO‐F, suggesting faster overall interfacial relaxation under NitRR conditions. This shift indicated that the ordered 1D hexagonal mesopores in FeSAs@*m*UiO‐P effectively facilitated charge transfer and reduced polarization resistance during the nitrate reduction process. In contrast, under HER conditions, both catalysts exhibited nearly identical Bode‐phase peaks, implying similar charge‐transfer behavior in the simple proton reduction process. These results were further supported by the DRT analysis (Figure 3f). Under NitRR conditions, both catalysts exhibited distinct relaxation peaks in the range of τ ≈ 10^−4^–10^−2^ s, which were tentatively associated with overlapping nitrate‐related interfacial processes involving charge transfer, adsorbate relaxation, and proton‐coupled reactions. FeSAs@*m*UiO‐P showed these peaks at shorter relaxation times compared with FeSAs@*m*UiO‐F, suggesting more rapid interfacial kinetics. By contrast, under HER conditions, both catalysts exhibited comparable peaks in the τ ≈ 10^−4^–10^−3^ s range, indicating similar charge‐transfer behavior in the absence of nitrate reduction. Collectively, these observations suggest that the geometry‐tailored mesoporous structure and the associated interfacial water environment may preferentially facilitate nitrate‐reduction‐related interfacial processes, while having a less pronounced effect on the HER‐related response.

### 2.4 Geometric Frustration of Interfacial Water for Enhanced Proton Supply

To elucidate the reaction pathway and identify key intermediates, *operando* ATR‐FTIR spectroscopy was performed for both FeSAs@*m*UiO‐P (Figure 4a,b) and FeSAs@*m*UiO‐F (Figure 4c,d). As shown in Figure 4a,c, upon applying potentials ranging from OCP to −1.4 V versus RHE, distinct vibrational bands were observed, which could be assigned to different NitRR intermediates [44]. These results suggested a reaction pathway involving the stepwise conversion of NO_3_^−^ into NO_2_^−^ and eventually NH_3_. The spectra also exhibited a clear potential‐dependent evolution. As the applied potential became more negative, the intensity of all detectable intermediate bands increased, reflecting enhanced activation of nitrate and accelerated formation of surface intermediates under stronger reducing conditions. In addition, a band at ∼1630 cm^−1^ was attributed to the O─H bending vibration of water, whose intensity increased with applied potential, reflecting enhanced water adsorption [45]. It should be noted that the nitrate‐related FTIR band reflected the population of interfacial adsorbed nitrate species rather than the absolute nitrate concentration in the bulk electrolyte. Therefore, an increase in the nitrate‐related signal under cathodic polarization may arise from the continuous adsorption and local enrichment of nitrate at the catalyst/electrolyte interface, even while nitrate reduction was simultaneously occurring. For FeSAs@*m*UiO‐F, the in situ FTIR spectra revealed only a clear and well‐defined *NO_2_ absorption peak, with an intensity comparable to that observed for the P‐type catalyst. In contrast, the characteristic bands corresponding to other key reaction intermediates, such as *NO, *NH_2_OH, and *NH_3_, were barely detectable and remained weaker throughout the applied potential range. Together with the previously observed isotopic shifts and electrochemical results, this suggested that although *NO_2_ could be generated on the FeSAs@*m*UiO‐F surface, its subsequent hydrogenation steps might be less favorable, potentially resulting in the accumulation of *NO_2_ and reduced progression along downstream reaction pathways. The relatively weak signals of later‐stage intermediates may reflect less efficient proton‐coupled electron‐transfer processes and limited conversion toward NH_3_, which could partly account for the lower FE of FeSAs@*m*UiO‐F. To further probe consumption of NO_3_^−^, in situ Raman spectroscopy was conducted (Figure 4e). The characteristic band of NO_3_^−^ at 1050 cm^−1^ gradually decreased with increasing applied potential, confirming the progressive activation and conversion of NO_3_^−^ during catalysis.

**FIGURE 4:**
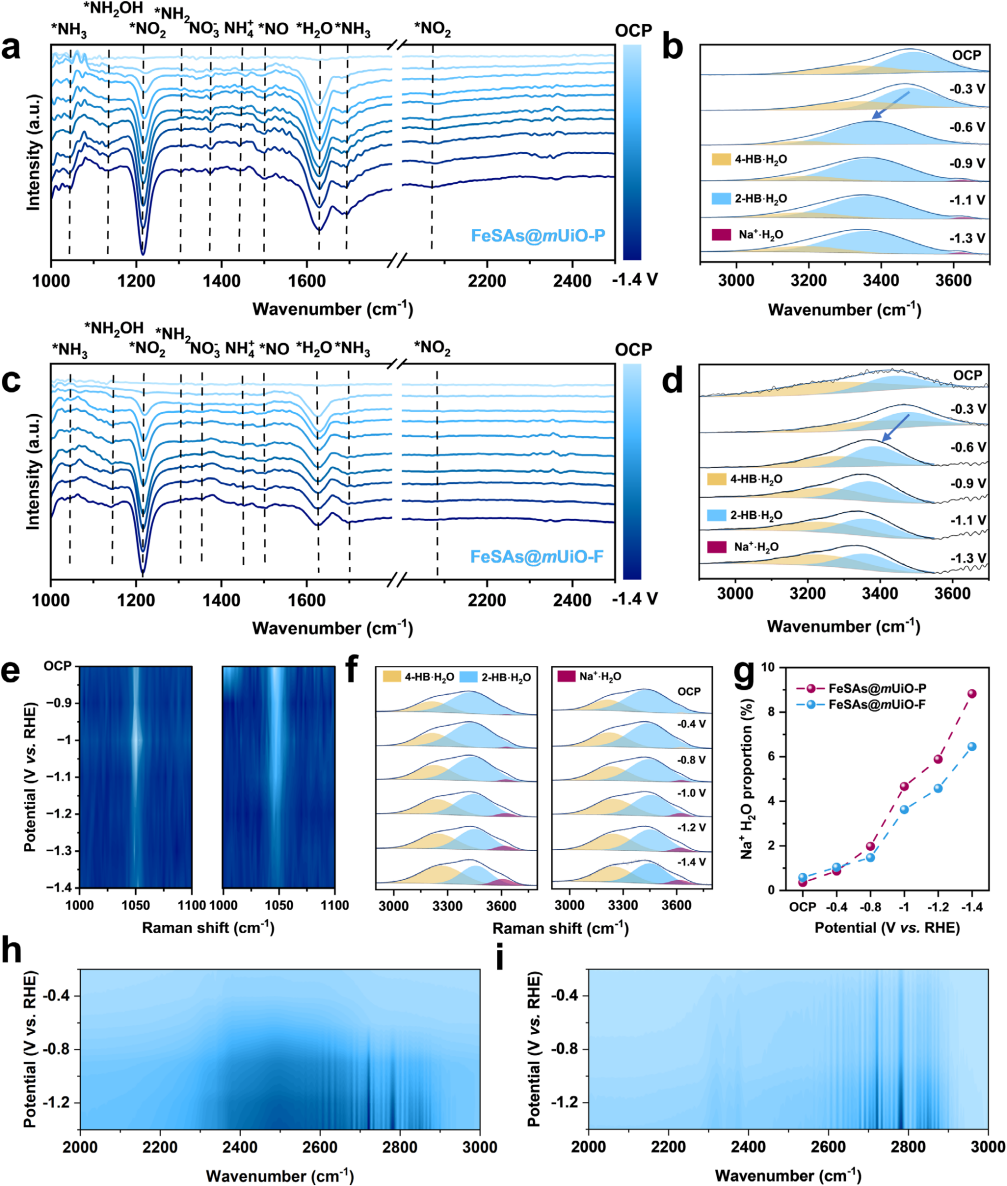
Geometry‐induced interfacial water frustration and enhanced proton supply. (a–d) In situ FTIR spectra of (a, b) FeSAs@*m*UiO‐P and (c, d) FeSAs@*m*UiO‐F. (e) In situ Raman spectra of FeSAs@*m*UiO‐P (left) and FeSAs@*m*UiO‐F (right). (f) In situ Raman spectra of interfacial water of FeSAs@*m*UiO‐P (left) and FeSAs@*m*UiO‐F (right). (g) Comparison of the proportion of Na^+^·H_2_O of FeSAs@*m*UiO‐P and FeSAs@*m*UiO‐F under different potentials. (h, i) In situ FTIR spectra of FeSAs@*m*UiO‐P and FeSAs@*m*UiO‐F in D_2_O electrolyte, respectively.

To examine whether this behavior was associated with the reorganization of interfacial water, Gaussian fitting was performed on the broad O─H stretching band (2900–3700 cm^−1^) [46, 47]. This broad band could be deconvoluted into three components: 4‐coordinated hydrogen‐bonded water (4‐HB·H_2_O), 2‐coordinated hydrogen‐bonded water (2‐HB·H_2_O), and Na^+^‐hydrated water (Na^+^·H_2_O), corresponding to strongly hydrogen‐bonded water, weakly hydrogen‐bonded water, and non‐hydrogen‐bonded (“free”) water, respectively. As shown in Figure 4b,d, both catalysts exhibited an obvious red‐shift of the strong and weak hydrogen‐bonded water peaks with increasingly negative potentials, which reflected enhanced polarization of water molecules and elongation of O─H bonds. Moreover, the free‐water peak (∼3600 cm^−1^) emerged exclusively for FeSAs@*m*UiO‐P at potentials more negative than −0.9 V versus RHE. Owing to its weakest hydrogen bonding, free‐water‐like species may represent a more labile interfacial water population available for proton‐involved surface reactions. This spectral change occurred within the potential region where the FE increased markedly beyond −0.9 V versus RHE. Collectively, these features indicate a potential‐dependent reorganization of the interfacial water environment. The hydrogen‐bonded components may support proton transfer through the interfacial water network, whereas the increasing non‐hydrogen‐bonded water contribution may represent a more labile water population available for proton‐involved surface reactions. Interestingly, the free‐water band appeared only in the hexagonal mesopores of FeSAs@*m*UiO‐P and was absent in the cubic mesopores of FeSAs@*m*UiO‐F. Based on these potential‐dependent spectral differences, we propose that the 1D channels of hexagonal mesopores favor a more ordered hydrogen bonding, thereby facilitating the exposure of non‐hydrogen‐bonded molecules. In contrast, the 3D cubic mesopores induce a disordered organization of water molecules with isotropic weak hydrogen bonding, showing a lower free‐water‐related spectral contribution and consequently restricting the hydrogenation process of FeSAs@*m*UiO‐F due to limited proton transfer.

Complementary in situ Raman measurements were also performed (Figure 4f). The difference between the FTIR and Raman observations may reflect their different selection rules and sensitivities to interfacial water populations. Unlike the FTIR spectra, all three water‐related components were observed for both catalysts across the entire potential range. To compare their hydrogenation abilities, the relative fractions of different water species were quantified by calculating the area ratios of each deconvoluted sub‐band to the total O─H envelope. As shown in Figure 4g, FeSAs@*m*UiO‐P displayed a comparable fraction of free‐water‐like species to FeSAs@*m*UiO‐F at low potentials. However, once the potential exceeded −1.0 V versus RHE, a clear contrast emerged between the two catalysts, consistent with the threshold‐like behavior of FeSAs@*m*UiO‐P observed at −0.9 V versus RHE in the FTIR spectra. This further supported a difference in the potential‐dependent interfacial water response enabled by 1D mesopore confinement.

To further probe the involvement of interfacial water species, H/D isotope‐exchange FTIR measurements were conducted by replacing H_2_O with D_2_O (Figure 4h,i). Upon isotope exchange, the O─H vibrational feature in the high‐wavenumber region shifted to the characteristic O–D region at approximately 2500 cm^−1^, confirming the participation of water species under reaction conditions. In addition, FeSAs@*m*UiO‐P exhibited a more pronounced O–D vibrational response than FeSAs@*m*UiO‐F, suggesting a greater extent of hydrogen incorporation during nitrate reduction. This observation was consistent with the enhanced hydrogenation behavior observed for FeSAs@*m*UiO‐P and further supported a more favorable proton‐involved reaction process in the hexagonal mesoporous architecture.

### 2.5 *Operando* Observation of Potential‐Dependent Active‐Site Reconstruction

To interrogate the atomic‐scale origin of the threshold behavior observed in NitRR kinetics, *operando* x‐ray absorption spectroscopy (XAS) was performed on both FeSAs@*m*UiO‐P (Figure 5a–c) and FeSAs@*m*UiO‐F (Figure 5d–f). The normalized potential‐resolved Fe K‐edge XANES spectra are presented in Figure 5a,d together with those of reference compounds. At open circuit and potentials more positive than −0.8 V versus RHE, both catalysts exhibited spectra closely resembling that of Fe_2_O_3_, indicating that the Fe centers predominantly existed in the Fe^3+^ oxidation state. Upon applying more negative potentials, the absorption edges of both catalysts gradually shifted toward lower energies. At −1.1 V versus RHE, the Fe K‐edge positions of both catalysts were shifted toward lower energies relative to their OCP states, indicating the partial reduction of Fe^3+^ species under nitrate reduction conditions. Notably, the edge position of FeSAs@*m*UiO‐P was located between those of FeO and Fe foil, whereas that of FeSAs@*m*UiO‐F remained between Fe_2_O_3_ and FeO. A greater extent of Fe reduction was therefore indicated for FeSAs@*m*UiO‐P, suggesting that Fe sites with a lower average oxidation state were generated in the hexagonal mesoporous architecture. Upon removal of the applied potential, the XANES spectrum of FeSAs@*m*UiO‐P was largely restored to its initial state, whereas that of FeSAs@*m*UiO‐F remained partially shifted relative to the pristine catalyst, indicating a greater post‐potential recovery in the hexagonal mesoporous architecture.

**FIGURE 5:**
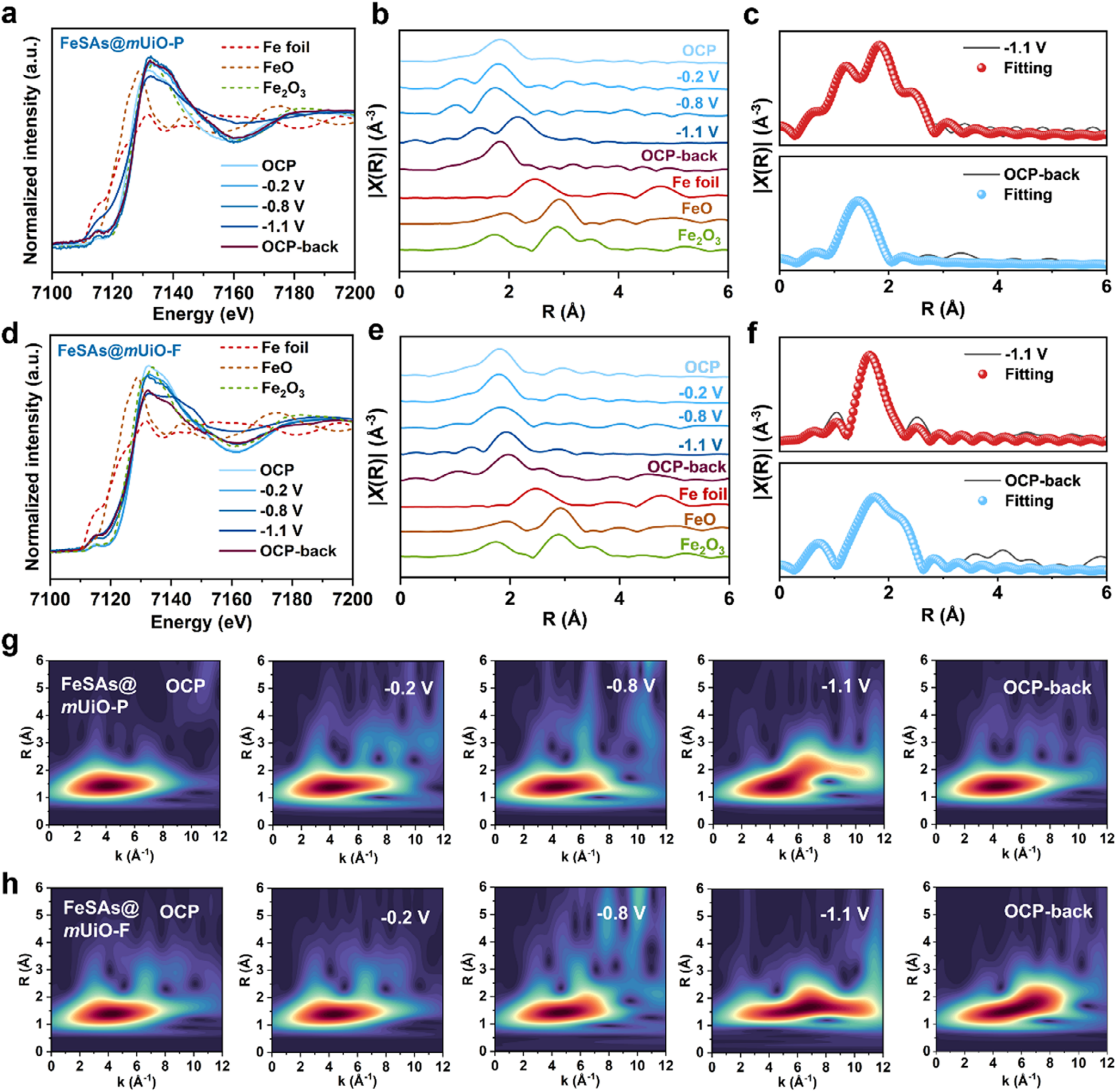
Potential‐dependent evolution of the average Fe coordination environment during NitRR. (a, d) In situ Fe K‐edge XANES spectra, (b, e) FT‐EXAFS spectra, (c, f) EXAFS fittings, and (g, h) WT spectra of FeSAs@*m*UiO‐P and FeSAs@*m*UiO‐F under different potentials.

The corresponding *operando* FT‐EXAFS spectra further supported this conclusion (Figure 5b,e). At open circuit and relatively positive potentials, both catalysts displayed a dominant Fe─O scattering peak that closely resembled that of the Fe_2_O_3_ reference. As the potential became more negative, the main Fe─O peak gradually shifted toward larger apparent *R* values, accompanied by changes in the higher‐shell region, indicating a potential‐induced reconstruction of the local Fe coordination environment. Notably, a substantially larger *R*‐space shift was observed for FeSAs@*m*UiO‐P than for FeSAs@*m*UiO‐F at −1.1 V versus RHE, suggesting a more extensive reorganization of the Fe coordination structure in the hexagonal mesoporous architecture. Upon removal of the applied potential, the FT‐EXAFS spectrum of FeSAs@*m*UiO‐P largely recovered to its initial Fe─O‐dominated state, whereas that of FeSAs@*m*UiO‐F retained discernible deviations from the pristine spectrum, indicating only partial recovery of the reconstructed Fe species. These observations suggested that both catalysts underwent dynamic structural evolution under nitrate reduction conditions, although the extent of the coordination changes and the degree of post‐potential recovery differed between the two catalysts.

To further clarify the structural evolution of the Fe centers under operating potentials, quantitative EXAFS fitting was performed in the first‐shell fitting range (*R* = 1–3 Å), and the best‐fit parameters are summarized in Figures 5c,f and S34–S35 and Tables S9 and S10. Due to the phase‐shift effect in EXAFS, the FT‐EXAFS peak positions are typically observed at *R* values approximately 0.3 Å shorter than the actual interatomic distances. At open circuit and relatively positive potentials, the Fe K‐edge EXAFS spectra of both FeSAs@*m*UiO‐P and FeSAs@*m*UiO‐F could be satisfactorily fitted using a single Fe─O shell, giving an average coordination number of ∼4.5 and a Fe─O bond length of ∼2.00 Å. These results were consistent with atomically dispersed Fe─O_4_‐like sites anchored on the UiO‐66 framework, confirming that both catalysts initially contained isolated Fe species. Upon applying more negative potentials, pronounced reconstruction of the local Fe coordination environment occurred in both catalysts. In addition to the first‐shell Fe─O contribution, an additional higher‐shell Fe‐associated scattering contribution was required to achieve satisfactory fitting, consistent with the development of an Fe‐associated higher‐shell contribution under nitrate reduction conditions, which was modeled using an Fe─O─Fe‐like scattering path. For clarity, this spectroscopically inferred working‐state environment is hereafter referred to as a more highly associated Fe─O─Fe‐like motif. For FeSAs@*m*UiO‐P, the Fe─O coordination number increased to approximately 6, accompanied by a pronounced higher‐shell contribution. In contrast, FeSAs@*m*UiO‐F exhibited a lower Fe─O coordination number of approximately 4.8–5 together with a weaker higher‐shell contribution. These results demonstrate that both catalysts undergo potential‐induced Fe‐site association, while the resulting reconstruction modes are markedly different. The more pronounced Fe‐associated higher‐shell contribution of FeSAs@*m*UiO‐P may reflect a greater contribution from neighboring or oxygen‐bridged Fe environments, which could support cooperative interactions with nitrate‐derived intermediates. FeSAs@*m*UiO‐F exhibited a weaker higher‐shell contribution, indicating a smaller average change in its Fe coordination environment. These distinct potential‐dependent coordination responses coincided with the different nitrate‐reduction performances of the two catalysts.

Remarkably, the onset potential for Fe‐site reconstruction coincides with the potential region where a pronounced increase in free‐water‐related species is observed by *operando* vibrational spectroscopy. At the same potential range, *operando* FTIR measurements reveal the emergence of substantially stronger downstream hydrogenated intermediates on FeSAs@*m*UiO‐P, whereas FeSAs@*m*UiO‐F exhibits more evident accumulation of *NO_2_‐related species and much weaker signals from subsequent hydrogenation products. These observations suggest that the hexagonal mesoporous architecture provides a local reaction environment that favors continuous hydrogenation during nitrate reduction. Recent studies have shown that hydrogen adsorption and hydrogenation‐related surface processes can induce dynamic restructuring of isolated metal sites under reductive conditions [48, 49]. In this context, the more efficient hydrogenation behavior observed on FeSAs@*m*UiO‐P may provide favorable conditions for Fe‐site evolution, coinciding with the development of a highly associated Fe─O─Fe motif. In contrast, the less efficient hydrogenation process on FeSAs@*m*UiO‐F is accompanied by the formation of a low‐associated Fe─O─Fe motif under otherwise identical reaction conditions. Importantly, after removal of the applied potential, the Fe‐associated scattering features largely disappear and the spectra recover toward their initial Fe─O coordination environments, supporting substantial post‐potential recovery of the average Fe coordination environment. These results suggest that nitrate reduction proceeds through a dynamic evolution of Fe active sites that is strongly influenced by the mesopore‐regulated local reaction environment. The interplay between intermediate hydrogenation and reversible Fe‐site reconstruction may contribute to the different catalytic responses of the two mesoporous architectures.

In situ WT analysis of the Fe K‐edge EXAFS was further employed to monitor the evolution of the local Fe coordination environment under operating potentials (Figure 5g,h). At OCP, −0.2 V, and −0.8 V versus RHE, both FeSAs@*m*UiO‐P and FeSAs@*m*UiO‐F exhibit a dominant intensity maximum centered at *k* ≈ 6–7 Å^−1^ and *R* ≈ 1.5–2.0 Å, which is characteristic of Fe─O scattering and consistent with the initial Fe─O_4_‐like configuration. Upon applying −1.1 V versus RHE, an additional high‐k scattering feature emerges in both catalysts, accompanied by a broadening of the main WT contour toward larger R values, indicating the formation of Fe‐associated higher‐shell scattering contributions under reductive conditions. Notably, the WT evolution is more pronounced for FeSAs@*m*UiO‐P, which exhibits a stronger and more distinct high‐k feature at −1.1 V versus RHE. In contrast, FeSAs@*m*UiO‐F shows a comparatively weaker and more diffuse high‐k contribution, suggesting a lower degree of Fe‐site association. These observations are consistent with the EXAFS fitting results, which indicate the formation of a highly associated Fe─O─Fe motif in FeSAs@*m*UiO‐P and a low‐associated Fe─O─Fe motif in FeSAs@*m*UiO‐F. After removal of the applied potential, the WT features of both catalysts largely recover toward their initial Fe─O‐dominated patterns, supporting substantial post‐potential recovery of the average Fe coordination environment. The WT analysis therefore provides spectroscopic evidence for potential‐dependent changes in Fe‐associated scattering in both catalysts. These changes are consistent with the development of more pronounced Fe─O─Fe‐like coordination in FeSAs@*m*UiO‐P and a less extensive response in FeSAs@*m*UiO‐F.

To further evaluate the reversibility of the reconstructed Fe species, XAS measurements were performed after drying the catalysts under vacuum (Figures S36 and S37). For both FeSAs@*m*UiO‐P and FeSAs@*m*UiO‐F, the XANES, EXAFS, and WT features largely recovered to those of the initial Fe─O_4_‐like state, indicating that the Fe‐associated motifs generated under cathodic polarization were not permanently stabilized species. Instead, they could approach the initial Fe─O‐dominated coordination state upon removal of the reaction conditions. Furthermore, after 10 h of continuous nitrate reduction, FeSAs@*m*UiO‐P retained an Fe─O‐dominated average coordination environment, while no Fe nanoparticles or aggregates were detected in the examined STEM regions within the resolution of the measurement (Figures S38 and S39). These results support the resistance of the catalyst to irreversible Fe aggregation and substantial recovery of its average coordination environment.

Based on the *operando* spectroscopic trends, we propose the structural interpretation illustrated in Figure 6a as a schematic working hypothesis that integrates the average spectroscopic observations, rather than as a uniquely resolved atomistic reconstruction pathway. At open circuit, both catalysts contain atomically dispersed isolated Fe─O_4_ sites anchored within the UiO‐66 framework. Upon applying cathodic potentials, the Fe centers undergo partial reduction and dynamically evolve toward Fe─O─Fe‐associated multinuclear motifs through oxygen‐bridged interactions between neighboring Fe sites. However, the extent of this reconstruction strongly depends on the mesoporous architecture. In FeSAs@*m*UiO‐P, the Fe sites evolve into a highly associated Fe─O─Fe motif, whereas FeSAs@*m*UiO‐F exhibits only a partially associated reconstruction state. The more extensive Fe‐site association in FeSAs@*m*UiO‐P generates a larger population of neighboring Fe centers and oxygen‐bridged configurations, which facilitate intermediate stabilization and promote successive hydrogenation steps during nitrate reduction. In contrast, the less‐associated motif formed in FeSAs@*m*UiO‐F provides fewer cooperative Fe environments and therefore exhibits lower hydrogenation activity. Importantly, after removal of the applied potential, both reconstructed states largely revert toward the initial Fe─O_4_ coordination environment, supporting substantial recovery toward the initial Fe─O‐dominated environment after removal of the applied potential. Therefore, the superior NitRR performance of FeSAs@*m*UiO‐P may arise in part from its ability to form a more highly associated and reversible Fe─O─Fe motif under operating conditions, while maintaining the atomically dispersed configuration after the reaction. Accordingly, the proposed sequence illustrates one interpretation consistent with the experimental observations, while the direction of causality between water reorganization and Fe‐site evolution remains unresolved.

**FIGURE 6:**
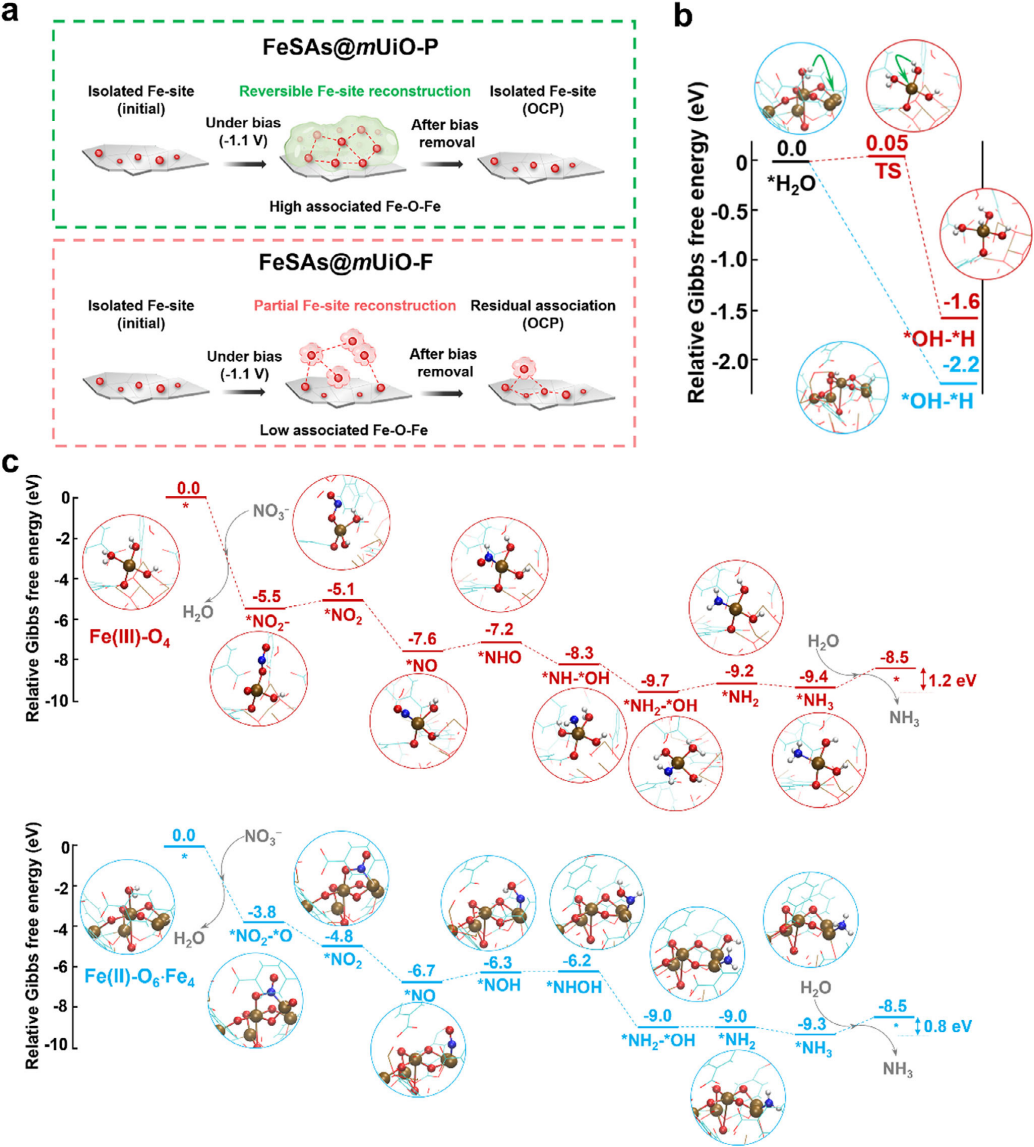
(a) Proposed structural interpretation of the potential‐dependent Fe‐site evolution during NitRR. (b) Calculated free‐energy profiles for water activation on the two systems, ^T^FeO_4_ (red) and ^O^FeO_6_·Fe_4_ (blue). Green arrows indicate hydrogen atom transfer. Free energies (in eV) are evaluated at 298 K using the computational hydrogen electrode method. Iron, oxygen, nitrogen, and hydrogen are shown in brown, red, blue, and white, respectively. (c) Calculated free‐energy profiles for nitrate reduction to ammonia on Fe‐containing motifs embedded in UiO‐66(Zr): (red) mononuclear ^T^Fe site initially coordinated in a tetrahedral FeO_4_ environment and (blue) multinuclear ^O^FeO_6_·Fe_4_ system, where an octahedrally coordinated ^O^Fe center is connected to four Fe sites through shared oxygen ligands.

### 2.6 Calculated Energetics of Enhanced Activity on Evolved Fe─O─Fe Motifs

Water activation on both Fe‐containing motifs is thermodynamically favorable and should proceed readily (Figure 6b). Here, ^T^Fe and ^O^Fe denote Fe centers that are tetrahedrally and octahedrally coordinated by oxygen atoms in the initial structures.The reaction to form *H and *OH is strongly exergonic, with free‐energy changes of −1.6 eV on the ^T^Fe site and −2.2 eV on the multinuclear system. On the mononuclear ^T^Fe site, both *H and *OH remain bound to the same Fe center, and the reaction proceeds with a small but finite activation barrier ∼0.05 eV. In contrast, water dissociation on the multinuclear system is barrierless and exhibits clear spatial separation of the products: *OH is stabilized on the ^O^Fe site, while *H adopts a bridging configuration between two neighboring Fe atoms.

Figure 6c presents the calculated free‐energy profiles for nitrate reduction to ammonia on two Fe‐containing motifs embedded in UiO‐66(Zr): a mononuclear ^T^FeO_4_ site and a multinuclear ^O^FeO_6_·Fe_4_ motif [31, 50, 51]. In the ^O^FeO_6_·Fe_4_ motif, the ^O^Fe center is connected to four Fe atoms through shared oxygen ligands, and the ^O^FeO_6_·Fe_4_ structure is used as a representative oxygen‐bridged multinuclear motif rather than a structurally defined Fe_4_ cluster. While both environments are capable of promoting nitrate reduction, their catalytic behaviors differ due to the presence or absence of cooperative Fe centers, resulting in different energetic spans. Nitrate activation on both catalysts begins with ligand exchange between NO_3_^−^ and pre‐adsorbed water molecules at the Fe sites, followed by spontaneous N─O bond cleavage to form co‐adsorbed *NO_2_ and *O species. On the ^T^Fe site, both *NO_2_ and *O remain bound to the same ^T^Fe center, leading to significant local crowding and strong competition for coordination. In contrast, on the multinuclear motif, the dissociation products are spatially separated: *NO_2_ adopts a bidentate binding configuration, while the dissociated O atom migrates to and is stabilized by one of the adjacent Fe sites. This cooperative adsorption effectively lowers the energetic penalty in the next step (from *NO_2_‐*O to *NO_2_). Specifically, this step is thermodynamically disfavored on the ^T^Fe site, with a positive free‐energy change of ∼0.4 eV, whereas it is exergonic on the multinuclear system.

In the subsequent steps, the reduction pathways on the two representative structural models are similar. Hydrogenation proceeds through the sequence *NO → *NHO (or *NOH) → *NH‐*OH (or *NHOH) → *NH_2_‐*OH → *NH_2_ → *NH_3_ → NH_3_(g) + *. Notably, after the initial *NO formation in the multinuclear system, the subsequent hydrogenation steps occur on a Fe site adjacent to the °Fe center, indicating their functional separation.

The better performance of the multinuclear motif is further highlighted by state‐determining analysis [52]. On the ^T^Fe site, the lowest‐energy adsorbed intermediate is *NH_2_‐*OH at −9.7 eV, while NH_3_(g) is at −8.5 eV, resulting in a large energetic span of 1.2 eV that limits catalytic efficiency. In contrast, on the multinuclear motif, the lowest‐energy state corresponds to *NH_3_ at −9.3 eV, with NH_3_(g) again at −8.5 eV, yielding a substantially smaller energetic span (0.8 eV). This reduced span directly translates into a faster kinetics for ammonia formation. Taken together, these results demonstrate that, while both motifs are active, the multinuclear °FeO_6_·Fe_4_ motif achieves better catalytic efficiency by avoiding site crowding and offering a kinetic advantage over the mononuclear ^T^Fe site.

## 3 Conclusion

In summary, this study identifies a potential‐dependent correspondence among mesopore architecture, interfacial water organization, and Fe‐site evolution during electrochemical nitrate reduction. The 1D hexagonal channels of FeSAs@*m*UiO‐P are associated with a larger free‐water‐related spectral contribution and an interfacial environment favorable for proton‐involved hydrogenation. Operando XAS further reveals potential‐dependent changes in the average Fe coordination environment of both catalysts, with FeSAs@*m*UiO‐P exhibiting a more pronounced Fe‐associated higher‐shell contribution than its cubic counterpart. These interfacial and structural differences coincide with the higher NH_3_ selectivity and yield rate of FeSAs@*m*UiO‐P, which achieves a FE of 99.1% and an NH_3_ yield rate of 182 µmol h^−1^ cm^−2^ at −1.05 V versus RHE. Together, these findings show that the catalytic behavior of Fe single sites in mesoporous frameworks is closely connected to their reaction‐state interfacial and coordination environments. The correlated spectroscopic responses support a working hypothesis linking interfacial water reorganization to Fe‐site evolution, although the causal role of water remains to be established. This work highlights the importance of working‐state interface regulation in the design of nitrate‐reduction electrocatalysts and related proton‐coupled electrocatalytic systems.

## Author Contributions

The project was conceptualized by **X.L**., **Y.Z**., and **Y.Y**., with **Y.Z**. and **Y.Y**. providing the supervision. **X.L**. performed the catalyst synthesis. **X.L**., **N.C.‐R.C**., **K.C.‐W.W**., **A.S.N**., and **Y.A**. carried out the catalyst characterization and the related data processing. Electrochemical tests were performed by **X.L**., **H.N.N**., and **Q.M.P**. provided the calculation results. The manuscript was co‐written by **X.L**. and **Y.Z**. with the guidance of **Y.Y**. All authors participated in results discussion and provided comments on the manuscript.

## Conflicts of Interest

The authors declare no conflicts of interest.

## Supporting information




**Supporting File**: anie74835‐sup‐0001‐SuppMat.docx.

## Data Availability

The data that support the findings of this study are available from the corresponding author upon reasonable request.
