# Supplementary material for: Coordinating Mesopore Geometry and Interfacial Water Dynamics for Iron‐Site Evolution in Nitrate Electroreduction
Source: Angew Chem Int Ed Engl. 2026 Sep 19;65(45):e5865467. doi: 10.1002/anie.5865467 (PMC13725565; doi:10.1002/anie.5865467)
Supplement: Supplementary file 1 [file ANIE-65-e5865467-s001.docx]

**Supporting Information**

**Coordinating Mesopore Geometry and Interfacial Water Dynamics for Iron-Site Evolution in Nitrate Electroreduction**

*Xiangyang Liu,^#,a^ Yingji Zhao, ^#,*,a^* *Norman C.-R. Chen, ^#,a,b,c^ Kevin C.-W. Wu,^b,c,d,e^* *Ho Ngoc Nam,^a^ Quan Manh Phung,^f,g^* *Asep Sugih Nugraha,^h^ Yusuke Asakura,^a^ Yusuke Yamauchi*^a,h,i^*

a *Department of Materials Process Engineering, Graduate School of Engineering, Nagoya University, Nagoya, Japan*

*Email:* [*yingji.zhao@akane.waseda.jp*](mailto:yingji.zhao@akane.waseda.jp)

b *Molecular Science and Technology Program, Taiwan International Graduate Program, Academia Sinica, Taipei 10617, Taiwan*
c *International Graduate Program of Molecular Science and Technology (NTU-MST), National Taiwan University, Taipei 10617, Taiwan*
d *Department of Chemical Engineering, National Taiwan University, Taipei, 10617 Taiwan*
e *Center of Atomic Initiative for New Materials (AI-MAT), National Taiwan University, Taipei, 10617 Taiwan*
f *Department of Chemistry, Graduate School of Science, Nagoya University, Furo-cho, Chikusa-ku, Nagoya 464–8602, Japan.*
g *Institute of Transformative Bio-Molecules (WPI-ITbM), Nagoya University, Furo-cho, Chikusa-ku, Nagoya 464–8601, Japan*
h *Australian Institute for Bioengineering and Nanotechnology (AIBN), The University of Queensland, Brisbane, Queensland 4072, Australia*

*Email:* [*y.yamauchi@uq.edu.au*](mailto:y.yamauchi@uq.edu.au)

i *Department of Convergent Biotechnology and Advanced Materials Science, Kyung Hee University, 1732 Deogyeong-daero, Giheung-gu, Yongin-si, Gyeonggi-do, 17104, South Korea*

\# Xiangyang Liu, Yingji Zhao and Norman C.-R. Chen contributed equally to this work.

**Methods**

**Chemicals**

N, N-dimethylformamide (DMF), sodium nitrate (NaNO_3_), sodium sulfate (Na_2_SO_4_), sodium nitrite (NaNO_2_), ammonium chloride (NH_4_Cl), sodium hydroxide (NaOH), sulfamic acid, Nafion D-520 dispersion, acetic acid (AA), sulfuric acid (H_2_SO_4_), hydrochloric acid (HCl), N-(1-naphthyl) ethylenediamine dihydrochloride, sodium citrate, salicylic acid, hydrogen peroxide (H_2_O_2_), sodium hypochlorite and sulfanilic acid were purchased from FUJIFILM Wako Pure Chemical Corporation. Zirconium tetrachloride (ZrCl_4_), ferric chloride (FeCl_3_), sodium nitrate-^15^N (Na^15^NO_3_), Pluronic® F-127 (F-127), Synperonic® PE P105 (P-105) and sodium nitroferricyanide were obtained from Sigma-Aldrich Technologies. Tetrafluoroterephthalic acid (H_2_BDC-F_4_) was obtained from Tokyo Chemical Industry Co., Ltd. Carbon paper (TGPH060-1005) and Nafion Membrane (N117) were obtained from Moubic Co., Ltd. All chemicals were used without further purification.

**Synthesis of *m*UiO series**

50 mg P-105 was dispersed in 3 mL deionized water. Then, 60mg ZrCl_4_, 0.4 mL HCl, 0.6 mL AA were added into the solution. After 30 min stirring, 60 mg BDC-F_4_ was added. Then the mixture was heated at 60 °C for 12 h with stirring. The resulting powder was washed three times with DMF and then dried in a vacuum oven at 60 °C overnight. The product was denoted as UiO-66-P.

*m*UiO-F was synthesized with the same method except that P-105 was replaced with F-127.

**Synthesis of FeSAs@*m*UiO series**

100 mg synthesized *m*UiO-P was dispersed in 30 mL DMF. The powder was distributed uniformly after 30 min ultrasonication. Then 100 mg FeCl_3_ was added and the mixture was stirred at 80 °C for 24 h. After the reaction, the powder was collected by centrifugation and then washed with DMF three times. Then the powder was heated in a vacuum oven at 60 °C. The product was denoted as FeSAs@*m*UiO-P.

FeSAs@*m*UiO-F was synthesized with the same method except that *m*UiO-F was used for Fe loading.

**Materials Characterization**

Scanning Electron Microscopy (SEM, ZEISS Gemini 560) and Transmission Electron Microscopy (TEM, JEM-ARM200F) were used to study the morphology of all the materials. The XRD data were collected on a Smart Lab X-ray diffractometer in a 2θ range of 5-40° at a scan rate of 1° min^-1^ using Cu Kα radiation. N_2_ adsorption/desorption isotherms were measured using BELSORP MINI II at 77 K. The specific surface area was calculated using the Brunauer-Emmett-Teller (BET) model and pore size distribution was calculated using the Barrett-Joyner-Halenda (BJH) Model. PHI Quantes was used to carry out the XPS measurement with Al Kα radiation. Inductively coupled plasma optical emission spectroscopy (ICP-OES) measurements were conducted by a SPS 7800 Plasma Spectrometer (SII−Seiko Instruments Inc.).

**Electrochemical Measurements**

H-cell equipment was used for all the electrochemical measurements and its chambers were separated with Nafion 117 membrane. Nafion 117 membrane was first pretreated at 80 °C in 5% H_2_O_2_, DI water, 0.5 M H_2_SO_4_ and DI water, each for 1 h. All the electrochemical measurements were conducted in 0.5 M Na_2_SO_4_ with and without the addition of 0.1 M NaNO_3_. Concentration-dependent measurements were performed using 10, 100, and 500 mM nitrate, as indicated in the corresponding figure captions. The as-synthesized MOFs were used as working electrodes, a platinum coil was used as counter electrode and Ag/AgCl in saturated KCl solution was used as reference electrode. The applied potentials were converted to reversible hydrogen electrode (RHE), according to the Nernst equation:

$$E_{RHE}=E_{Ag/AgCl}+0.059*pH+0.197$$

where a pH value of 7 is used. Electrochemical measurements were performed using the electrochemical workstation at room temperature and atmospheric pressure. Before testing, the electrolyte was first purged with Ar gas for 15 min.

For the electrode preparation, the catalysts were coated on the carbon paper using the drop-casting technique. First, 6 mg of catalysts was dispersed in the mixture of 980 μL of ethanol and 20 μL of 5% Nafion solution. The ink was then sonicated for over 30 min to obtain a uniform dispersion, and further dropped on the carbon paper with the addition amount of 0.96 mg cm^-2^.
**Determination of ammonia**

Ammonia concentration was quantified using the indophenol blue method. Briefly, a color reagent was prepared by dissolving NaOH (2 g), sodium citrate (2.889 g), and salicylic acid (2.889 g) in 50 mL of deionized water. An aliquot of the electrolyte (40 μL) was diluted with 1.96 mL of 0.5 M Na_2_SO_4_ and subsequently mixed with 2 mL of the freshly prepared color reagent. The resulting solution was then reacted with 1 mL of 0.05 M sodium hypochlorite and 200 μL of 1.0 wt% sodium nitroferricyanide. After incubation in the dark for 2 h, the UV–vis absorbance of the solution was measured at 655 nm to determine the ammonia concentration.

**Determination of nitrite**

Nitrite concentration was determined using a colorimetric method. The color reagent was prepared by dissolving sulfanilic acid (500 mg) in a mixture of deionized water (95 mL) and acetic acid (5 mL), followed by sonication for 5 min. Subsequently, N-(1-naphthyl) ethylenediamine dihydrochloride (5 mg) was added and fully dissolved. For UV–vis analysis, 200 μL of the electrolyte was diluted with 800 μL of 0.5 M Na_2_SO_4_ to adjust the concentration into the detection range, after which 4 mL of the freshly prepared color reagent was added. The mixture was incubated in the dark for 15 min, and the nitrite concentration was quantified by measuring the absorbance at 540 nm.

**Determination of nitrate**

Nitrate concentration was quantified by UV–vis spectroscopy. An aliquot of the electrolyte (0.1 mL) was diluted with 4.9 mL of 0.5 M Na_2_SO_4_ to bring the concentration into the detection range. Subsequently, 0.1 mL of 1 M HCl and 0.01 mL of a 0.8 wt% sulfamic acid solution were added to eliminate nitrite interference. The mixture was incubated in the dark for 15 min, after which the UV–vis spectrum was recorded. The nitrate absorbance was calculated using the following equation:

$$A=A_{220nm}-2A_{275nm}$$

where A_220nm_ and A_275nm_ correspond to the absorbance values at 220 and 275 nm, respectively.

**^15^N isotope-labeling experiment**

An isotope-labeling experiment was conducted to identify the nitrogen source of the produced ammonia. Electrolysis was performed using an electrolyte composed of 0.5 M Na_2_SO_4_ and 0.1 M Na^15^NO_3_ (98 atom% ^15^N). The electroreduction of ^15^NO_3_^-^ was carried out at −1.05 V (vs. RHE) for 0.5 h. The resulting ^15^NH_4_^+^ was subsequently analyzed by ^1^H nuclear magnetic resonance. The NMR measurement for ^14^NH_4_^+^ were identical to those used for ^15^NH_4_^+^.

**Calculation of FE and yield**

The Faradaic efficiency for NH_3_ production was calculated using the formula:

$${FE}_{NH_{3}}=\frac{8\times F\times C_{{NH}_{3}}\times V}{Q} \times100\%$$

The Faradaic efficiency for NO_2_^-^ production was calculated using the formula:

$${FE}_{{NO}_{2}^{-}}=\frac{2\times F\times C_{{NO}_{2}^{-}}\times V}{Q} \times100\%$$

The yield rate of NH_3_ was calculated using the formula:

$$r_{{NH}_{3}}=(C_{{NH}_{3}}\times V)/(t\times A)$$

Where F refers to the Faradaic constant 96485 (C mol^-1^) $C_{{NH}_{3}}$ (mol L^-1^) refers to the concentration of NH_3_; $C_{{NO}_{2}^{-}}$ (mol L^-1^) refers to the concentration of NO_2_^-^; V (L) refers to the volume of electrolyte in the cathode part; Q (C) refers to the total charge applied to the system; t (s) refers to the reaction time; A (cm^2^) refers to the reaction area of the electrode.

**Calculation of apparent turnover frequency**

The apparent turnover frequency for NH₃ production was calculated according to:

$$TOF=n_{{NH}_{3}}/(n_{Fe}\times t)$$

where $n_{{NH}_{3}}$ is the amount of NH_3_ produced during electrolysis, $n_{Fe}$ is the amount of Fe in the catalyst calculated from the catalyst loading and the Fe content determined by ICP-OES, and t is the electrolysis time. Because this calculation assumes that all Fe atoms are equally accessible and catalytically involved, the resulting values are reported as apparent Fe-normalized TOFs rather than intrinsic site-specific TOFs.

**Calculation of BET-area-normalized NH_3_ yield rate**
The BET-area-normalized NH₃ formation rate was then calculated as:

$$r_{{NH}_{3}, BET}= {r_{{NH}_{3}}}/{(S_{BET}} \times m_{loading})$$

Where $S_{BET}$ is the BET specific surface area of the catalyst, and $m_{loading}$ is the catalyst loading per unit geometric electrode area.

**Calculation of half-cell energy efficiency**

The estimated half-cell energy efficiency for NH₃ production was calculated according to:

$${EE}_{{NH}_{3}}=\frac{{(E}_{OER}^{0}-E_{{{NO}_{3}^{-}}/{{NH}_{3}}}^{0}) \times{FE}_{{NH}_{3}}}{{(E}_{OER}^{0}-E_{cathode})}\times100\%$$

where$E_{OER}^{0}$is the equilibrium potential of water oxidation (1.23 V vs. RHE), $E_{{{NO}_{3}^{-}}/{{NH}_{3}}}^{0}$ is the equilibrium potential of nitrate reduction to ammonia (0.69 V vs. RHE), $E_{cathode}$ is the applied cathodic potential versus RHE, and ${FE}_{{NH}_{3}}$is the Faradaic efficiency for NH₃ expressed as a fraction. This calculation assumes an ideal oxygen-evolution anode with zero anodic overpotential and does not include ohmic losses, membrane losses, electrolyte circulation, product separation, or other balance-of-plant energy requirements. The resulting values are therefore reported as estimated half-cell energy efficiencies rather than practical full-cell energy efficiencies.

**Computational details**

All density functional theory calculations were carried out using the OpenMX code interfaced with the Atomic Simulation Environment (ase).^1-5^ Models were built from the structure of UiO-66(Zr) optimized in our previous work.^6^ A linker-defective UiO-66(Zr) model was generated by removing one organic linker, and the resulting undercoordinated Zr sites were capped by H_2_O and OH groups.^7^ Two Fe-containing environments were considered. In the first model, one Fe atom and one H_2_O molecule were introduced into the defective region, forming a tetrahedrally coordinated FeO_4_ site through coordination with framework oxygen atoms. In the second model, one Fe atom was coordinated by framework oxygen atoms together with additional H_2_O and OH ligands, forming a distorted octahedral FeO_6_ site, after which four additional Fe atoms were added and bound to nearby oxygen atoms to generate a FeO_6_·Fe_4_ motif. All atomic positions were fully relaxed during geometry optimization. Periodic boundary conditions were applied in all three dimensions.

The generalized gradient approximation with the PBE functional was employed throughout.^8^ Norm-conserving pseudopotentials and pseudo-atomic orbitals supplied with OpenMX were used for all elements.^2^ For geometry optimization, the following pseudo-atomic orbital basis sets from the database Ver. 2019 were used: H5.0-s2 for H, C6.0-s2p2 for C, O6.0-s2p2 for O, N6.0-s2p2 for N, Fe5.5H-s2p2d1 for Fe, and Zr7.0-s3p2d1 for Zr. Dispersion interactions were included using the DFT-D3 method with Becke-Johnson damping.^9, 10^ An electronic temperature of 300 K was applied using Fermi-Dirac smearing. The real-space grid energy cutoff was set to 220 Ry. Due to the large size of the MOF cell (*a* ~ 21 Å), Brillouin zone sampling was restricted to the Γ-point. Self-consistent field convergence was achieved using the RMM-DIIS mixing scheme with an energy convergence threshold of 10^−7^ Hartree. Geometry optimizations were performed using the BFGS algorithm as implemented in ase until forces were smaller than 0.04 eV/Å. Single-point electronic energies were subsequently evaluated on the optimized geometries using larger basis sets to improve energetic accuracy, namely H6.0-s2p1 for H, C6.0-s2p2d1 for C, O6.0-s2p2d1 for O, N6.0-s2p2d1 for N, Fe5.5H-s3p2d1 for Fe, and Zr7.0-s3p2d2 for Zr, while all other computational parameters were kept unchanged. Harmonic vibrational frequency calculations were carried out for all adsorbed intermediates using finite-difference atomic displacements to obtain thermal free energy corrections. Free energies were evaluated at 298 K within the computational hydrogen electrode framework developed by Nørskov and co-workers, where the energy of a proton-electron pair equals one-half of an H_2_ molecule.^11^ The dissociation of water on the Fe sites was investigated using the climbing image nudged elastic band (CI-NEB) method to locate approximate transition states,^12^ which were subsequently refined using the Sella optimizer to converge to first-order saddle points characterized by a single imaginary vibrational frequency along the reaction coordinate.^13^

**Figure S1.** SAXS patterns of *m*UiO-F and FeSAs@*m*UiO-F. FeSAs@mUiO-F displayed characteristic scattering peaks at q = 0.572, 0.863, and 0.992 nm^−1^, which could be tentatively indexed to the (100), (110), and (111) reflections, respectively, assuming a *Pm*3̅*m* cubic mesostructure.

.

**Figure S2.** XRD patterns of *m*UiO-F and FeSAs@*m*UiO-F.

**Figure S3.** SEM image of FeSAs@*m*UiO-F. The scale bar represents 50 nm.

**Figure S4.** Low-resolution SEM images of (a) FeSAs@*m*UiO-P and (b) FeSAs@*m*UiO-F. The scale bar represents 400 nm.

**Figure S5**. Magnified TEM image of FeSAs@*m*UiO-P.

**Figure S6.** TEM image of FeSAs@*m*UiO-F. The scale bar represents 100 nm.

F

Fe

Zr

**Figure S7.** HR-STEM and EDS images of FeSAs@*m*UiO-F.

**Figure S8.** XPS spectra of the Fe 2p orbital of (a) FeSAs@*m*UiO-P and (b) FeSAs@*m*UiO-F.

**Figure S9.** XPS spectra of (a) C 1s, (b) O 1s and (c) F 1s orbitals of FeSAs@*m*UiO-P.

**Figure S10.** XPS spectra of (a) C 1s, (b) O 1s and (c) F 1s orbitals of FeSAs@*m*UiO-F.

**Figure S11.** (a) N_2_ isotherms and (b) pore size distribution of *m*UiO-P, *m*UiO-F, FeSAs@*m*UiO-P and FeSAs@*m*UiO-F.

**Figure S12.** Fe K-edge k^2^-weighted EXAFS oscillation curves of FeSAs@*m*UiO-P, FeSAs@*m*UiO-F with reference samples (Fe foil, FeO, Fe_2_O_3_).

**Figure S13.** EXAFS fittings of Zr K-edge of *m*UiO-P, *m*UiO-F, FeSAs@*m*UiO-P, and FeSAs@*m*UiO-F.

**Figure S14.** (a) The UV-visible absorption spectra and (b) corresponding concentration calibration curve for the determination of NO_3_^-^ in 0.5 M Na_2_SO_4_ aqueous solution.

**Figure S15.** (a) The UV-visible absorption spectra and (b) corresponding concentration calibration curve for the determination of NO_2_^-^ in 0.5 M Na_2_SO_4_ aqueous solution.

**Figure S16.** (a) The UV-visible absorption spectra and (b) corresponding concentration calibration curve for the determination of NH_4_^+^ in 0.5 M Na_2_SO_4_ aqueous solution.

**Figure S17.** FE for NH_3_, NO_2_^-^ and H_2_ production at different potentials for FeSAs@*m*UiO-P.

**Figure S18.** (a) NH_3_ and (b) H_2_ partial current density of FeSAs@*m*UiO-P and FeSAs@*m*UiO-F under different potentials.

**Figure S19.** (a) LSV curves of *m*UiO-P and *m*UiO-F in 0.5 M Na_2_SO_4_ and 0.1 M NaNO_3_ electrolyte. (b) Faradaic efficiency and (c) ammonium yield rate of *m*UiO-P and *m*UiO-F under different potentials.

**Figure S20.** LSV curves of FeSAs@*m*UiO-P and FeSAs@*m*UiO-F measured at nitrate concentrations of 10, 100, and 500 mM (a) without and (b) with iR compensation.

**Figure S21.** (a) Faradaic efficiency for NH_3_ production and (b) NH_3_ yield rates of FeSAs@*m*UiO-P and FeSAs@*m*UiO-F at different applied potentials in an electrolyte containing 500 mM nitrate.

**Figure S22.** Apparent Fe-normalized turnover frequencies of FeSAs@*m*UiO-P and FeSAs@*m*UiO-F at nitrate concentrations of (a) 100 and (b) 500 mM.

**Figure S23.** BET-surface-area-normalized NH_3_ yield rates of FeSAs@*m*UiO-P and FeSAs@*m*UiO-F at nitrate concentrations of (a) 100 mM and (b) 500 mM under different applied potentials.

**Figure S24**. (a) Linear sweep voltammetry curves, (b) potential-dependent Faradaic efficiencies for NH_3_ production, and (c) NH_3_ yield rates of FeSAs@*m*UiO-F samples with different Fe loadings.

**Figure S25.** ^1^H NMR spectra of electrolyte after NitRR with Na^14^NO_3_ and Na^15^NO_3_ as the N sources.

**Figure S26.** The UV-visible absorption spectra for determining the concentrations of ammonia in the electrolyte after electrolysis with and without 0.1 M nitrate.

**Figure S27.** Faradaic efficiency and ammonium yield rate of FeSAs@*m*UiO-P for 10 consecutive cycles at -1.05 V *vs.* RHE.

**Figure S28.** CV curves under different scan rates of (a) FeSAs@*m*UiO-P and (b) FeSAs@*m*UiO-F.

**Figure S29.** Plots of Δj/2 vs. scan rate, where Δj is the difference between the anodic and cathodic current density at +0.55 V vs. RHE in each CV curve extracted from the data shown in **Figure S28**.

**Figure S30.** *In situ* Nyquist plots under NitRR condition of (a) FeSAs@*m*UiO-P and (b) FeSAs@*m*UiO-F.

**Figure S31.** *In situ* Nyquist plots under HER condition of (a) FeSAs@*m*UiO-P and (b) FeSAs@*m*UiO-F.

**Figure S32.** *In situ* Bode plots under NitRR condition of (a) FeSAs@*m*UiO-P and (b) FeSAs@*m*UiO-F.

**Figure S33.** *In situ* Bode plots under HER condition of (a) FeSAs@*m*UiO-P and (b) FeSAs@*m*UiO-F.

**Figure S34.** FT-EXAFS fittings of FeSAs@*m*UiO-P under OCP.

**Figure S35**. FT-EXAFS fittings of FeSAs@*m*UiO-F under OCP.

**Figure S36.** (a) XANES spectra at the Fe K-edge, (b) FT-EXAFS spectra at the Fe K-edge, (c) EXAFS fittings and (d) WT of Fe K-edge of FeSAs@*m*UiO-P after the catalysis process and drying.

**Figure S37.** (a) XANES spectra at the Fe K-edge, (b) FT-EXAFS spectra at the Fe K-edge, (c) EXAFS fittings and (d) WT of Fe K-edge of FeSAs@*m*UiO-F after the catalysis process and drying.

**Figure S38.** (a) XANES spectra at the Fe K-edge, (b) FT-EXAFS spectra at the Fe K-edge, (c) WT of Fe K-edge of FeSAs@mUiO-P after 10 h of continuous electrolysis.

**Figure S39.** HR-STEM image of FeSAs@*m*UiO-P after 10 h of continuous electrolysis.

**Table S1.** The elemental content (wt%) of samples obtained from XPS.

| Samples | Fe (wt%) |
| --- | --- |
| FeSAs@*m*UiO-P | 2.0 |
| FeSAs@*m*UiO-F | 1.5 |

**Table S2.** Fe element proportion of samples determined by ICP measurement.

| Samples | Fe (wt%) |
| --- | --- |
| FeSAs@*m*UiO-P | 1.5 |
| FeSAs@*m*UiO-F | 1.2 |

**Table S3.** Surface areas and pore volumes of samples.

| Samples | Specific surface area  (m^2^ g^-1^) | Pore volume  (cm^3^ g^-1^) | Micropore volume  (cm^3^ g^-1^) | Mesopore volume (cm^3^ g^-1^) | Average pore size (nm) |
| --- | --- | --- | --- | --- | --- |
| *m*UiO-P | 986.53 | 0.6552 | 0.2952 | 0.3600 | 2.516 |
| *m*UiO-F | 536.79 | 0.3658 | 0.1067 | 0.2591 | 2.357 |
| FeSAs@*m*UiO-P | 440.43 | 0.4117 | 0.0990 | 0.3127 | 3.792 |
| FeSAs@*m*UiO-F | 369.36 | 0.3067 | 0.1447 | 0.1620 | 3.628 |

**Table S4.** Structural parameters extracted from the Fe K-edge EXAFS fitting.

| Sample | S_0_^2^ | CN | | R | E_0_ | R-factor |
| --- | --- | --- | --- | --- | --- | --- |
| FeSAs@*m*UiO-P | 0.95 | | 4.5 | 2.00 ± 0.02 | 1.438 | 0.009 |
| FeSAs@*m*UiO-F |  |  |  | 2.02 ± 0.02 | 1.118 | 0.009 |
| FeSAs@*m*UiO-P-Post-Vacuum-Dry |  |  |  | 1.97 ± 0.03 | 5.153 | 0.008 |
| FeSAs@*m*UiO-F-Post-Vacuum-Dry |  |  |  | 1.98 ± 0.02 | 2.588 | 0.007 |

**Table S5.** Structural parameters extracted from the Zr K-edge EXAFS fitting.

| Sample | S_0_^2^ | CN | | R | E_0_ | R-factor |
| --- | --- | --- | --- | --- | --- | --- |
| *m*UiO-P | 0.9 | | 4 | 3.56 ± 0.01 | 4.636 | 0.004 |
| *m*UiO-F |  |  |  | 3.56 ± 0.01 | 4.907 | 0.005 |
| FeSAs@*m*UiO-P |  |  |  | 3.54 ± 0.02 | 5.168 | 0.005 |
| FeSAs@*m*UiO-F |  |  |  | 3.57 ± 0.02 | 4.184 | 0.005 |

**Table S6.** Energy efficiency (EE) of FeSAs@*m*UiO-P and FeSAs@*m*UiO-F under different potentials.

| Potential (V vs. RHE) | EE, P (%) | EE, F (%) |
| --- | --- | --- |
| -0.8 | 20.08 | 19.50 |
| -0.9 | 23.70 | 21.68 |
| -1.0 | 23.85 | 20.46 |
| -1.05 | 23.47 | 20.42 |
| -1.1 | 21.21 | 19.47 |
| -1.15 | 20.03 | 19.38 |
| -1.2 | 19.51 | 17.42 |

**Table S7.** Comparison of Faradaic efficiency and ammonium yield rate of FeSAs@*m*UiO-P and other reported materials.

| Samples | Electrolysis | NH_3_ Faradaic efficiency | NH_3_ yield rate | Reference |
| --- | --- | --- | --- | --- |
| FeSAs@*m*UiO-P | 0.1 M NaNO_3_ + 0.5 M Na_2_SO_4_ | 99.1% | 214.86 mg h^-1^ mg_Fe_^-1^ | This work |
| Ru clusters | 1 M KOH + 1  M KNO_3_ | ~100% | 94.52 mg h^-1^ mg_cat_^-1^ | ^14^ |
| Fe SAC | 0.5 M KNO_3_ + 0.1 M K_2_SO_4_ | ~75% | 347.35 mg h^-1^ mg_Fe_^-1^ | ^15^ |
| Pd−CuO-200 | 0.1 M KNO_3_ + 1 M KOH | 90% | 71.4 mg h^-1^ mg_cat_^-1^ | ^16^ |
| Fe/Cu-NG | 0.1 M KNO_3_ + 1.0 M KOH | ~92.71% | 300.98 mg h^-1^ mg_SAC_^-1^ | ^17^ |
| Cu-*cis*-N_2_O_2_ | 1000 mg mL^−1^ N–KNO_3_ + 0.5 M Na_2_SO_4_ | ~86.43% | 138.09 mg h^-1^ mg_Cu_^-1^ | ^18^ |
| Cu–N–C | 0.1 M KNO_3_ + 0.1 M KOH | 84.7% | 212.5 mg h^-1^ mg_Cu_^-1^ | ^19^ |
| Ru_3%_-HEO | 0.01 M KNO_3_ + 0.1 M KOH | 91.3% | 282.44 mg h^-1^ mg_Ru_^-1^ | ^20^ |
| RuCu DAs/NGA | 0.1 M KNO_3_+0.1 M KOH | 95.7% | 125 mg h^-1^ mg_SAC_^-1^ | ^21^ |
| Cu_1_/WO_3_ | 22.5 mg L^−1^ of NO_3_^—^N + 0.05 M Na_2_SO_4_ | 93.7% | 1.55 mg h^-1^ mg_Cu_^-1^ | ^22^ |
| PdCuNiCoZn | 0.1M NaNO_3_ + 0.5 M Na_2_SO_4_ | 90.62% | 151.83 mg h^-1^ mg_cat_^-1^ | ^23^ |

**Table S8.** Comparison of the principal compositional, textural, electrochemical, and nitrate-reduction parameters of FeSAs@*m*UiO-P and FeSAs@*m*UiO-F.

| **Parameter** | **FeSAs@mUiO-P** | **FeSAs@mUiO-F** | **P/F comparison** |
| --- | --- | --- | --- |
| ICP Fe loading | 1.5 wt% | 1.2 wt% | 1.25× |
| XPS Fe content | 2.0 wt% | 1.5 wt% | 1.33× |
| BET surface area | 440.43 m^2^ g^-1^ | 369.36 m^2^ g^-1^ | 1.19× |
| Total pore volume | 0.4117 cm^3^ g^-1^ | 0.3067 cm^3^ g^-1^ | 1.34× |
| Micropore volume | 0.0990 cm^3^ g^-1^ | 0.1447 cm^3^ g^-1^ | 0.68× |
| Mesopore volume | 0.3127 cm^3^ g^-1^ | 0.1620 cm^3^ g^-1^ | 1.93× |
| Mesopore-volume fraction | 76.0% | 52.8% | +23.2 percentage points |
| Average pore size | 3.792 nm | 3.628 nm | 1.05× |
| C_dl_ | 280.15 μF cm^-2^ | 172.90 μF cm^-2^ | 1.62× |
| NH_3_ yield at −1.05 V (100 mM NO_3_^-^) | 182 μmol h^-1^ cm^-2^ | 151 μmol h^-1^ cm^-2^ | 1.21× |
| NH_3_ yield at −1.1 V (500 mM NO_3_^-^) | 282 μmol h^-1^ cm^-2^ | 201 μmol h^-1^ cm^-2^ | 1.40× |
| NH_3_ FE at −1.05 V (100 mM NO_3_^-^) | 99.1% | 85% | +14.1 percentage points |
| NH_3_ FE at −1.1 V (500 mM NO_3_^-^) | 88.22% | 79.36% | +8.86 percentage points |
| KIE | 1.58 | 1.83 | Lower for P |

**Table S9.** Structural parameters extracted from the *in situ* Fe K-edge EXAFS fitting of FeSAs@*m*UiO-P.

| Potential  (V *vs.* RHE) |  | S_0_^2^ | CN | R | E_0_ | R-factor |
| --- | --- | --- | --- | --- | --- | --- |
| OCP |  | 0.95 | 4.5 | 2.01 ± 0.01 | 4.273 | 0.003 |
| -1.1 | 1^st^ shell (Fe-O) | 0.95 | 6 | 2.12 ± 0.04 |  | 0.002 |
|  | 2^nd^ shell  (Fe-O-Fe) |  | 4.5 | 2.77 ± 0.03 |  |  |
| OCP-back |  | 0.95 | 4.5 | 1.98 ± 0.01 | -4.229 | 0.004 |

**Table S10.** Structural parameters extracted from the *in situ* Fe K-edge EXAFS fitting of FeSAs@*m*UiO-F.

| Potential  (V *vs.* RHE) |  | S_0_^2^ | CN | R | E_0_ | R-factor |
| --- | --- | --- | --- | --- | --- | --- |
| OCP |  | 0.95 | 4.5 | 1.99 ± 0.02 | -4.845 | 0.005 |
| -1.1 | 1^st^ shell  (Fe-O) | 0.55 | 4.8 | 2.02 ± 0.02 |  | 0.001 |
|  | 2^nd^ shell  (Fe-O-Fe) |  | 4 | 2.45 ± 0.07 |  |  |
| OCP-back | 1^st^ shell  (Fe-O) | 0.8 | 4.8 | 2.08 ± 0.01 |  | 0.005 |
|  | 2^nd^ shell  (Fe-O-Fe) |  | 4 | 2.45 ± 0.02 |  |  |

**References**

[1] Ozaki, T. Variationally optimized atomic orbitals for large-scale electronic structures. *Physical Review B* **67**, (2003): 155108.

[2] Ozaki, T.; Kino, H. Numerical atomic basis orbitals from H to Kr. *Physical Review B* **69**, (2004): 195113.

[3] Ozaki, T.; Kino, H. Efficient projector expansion for the ab initio LCAO method. *Physical Review B—Condensed Matter and Materials Physics* **72**, (2005): 045121.

[4] Lejaeghere, K., Bihlmayer, G., Björkman, T., Blaha, P., Blügel, S., Blum, V., *et al*. Reproducibility in density functional theory calculations of solids. *Science* **351**, (2016): aad3000.

[5] Larsen, A. H.; Mortensen, J. J.; Blomqvist, J.; Castelli, I. E.; Christensen, R.; Dułak, M.; Friis, J.; Groves, M. N.; Hammer, B.; Hargus, C. The atomic simulation environment—a Python library for working with atoms. *Journal of Physics: Condensed Matter* **29**, (2017): 273002.

[6] Shen, C.-H.; Zhao, Y.; Nam, H. N.; Zhu, L.; Phung, Q. M.; Austen, V.; Kim, M.; Jiang, D.; Wei, X.; Yokoshima, T. Unlocking coordination sites of metal–organic frameworks for high-density and accessible copper nanoparticles toward electrochemical nitrate reduction to ammonia. *Chemical Science* **16**, (2025): 7026.

[7] Liu, L.; Chen, Z.; Wang, J.; Zhang, D.; Zhu, Y.; Ling, S.; Huang, K.-W.; Belmabkhout, Y.; Adil, K.; Zhang, Y. Imaging defects and their evolution in a metal–organic framework at sub-unit-cell resolution. *Nature chemistry* **11**, (2019): 622-628.

[8] Perdew, J. P.; Burke, K.; Ernzerhof, M. Generalized gradient approximation made simple. *Physical Review Letters* **77**, (1996): 3865.

[9] Grimme, S.; Antony, J.; Ehrlich, S.; Krieg, H. A consistent and accurate ab initio parametrization of density functional dispersion correction (DFT-D) for the 94 elements H-Pu. *The Journal of Chemical Physics* **132**, (2010): 154104.

[10] Grimme, S.; Ehrlich, S.; Goerigk, L. Effect of the damping function in dispersion corrected density functional theory. *Journal of Computational Chemistry* **32**, (2011): 1456.

[11] Nørskov, J. K.; Rossmeisl, J.; Logadottir, A.; Lindqvist, L.; Kitchin, J. R.; Bligaard, T.; Jonsson, H. Origin of the overpotential for oxygen reduction at a fuel-cell cathode. The Journal of Physical Chemistry B 108, (2004): 17886-17892.

[12] Henkelman, G.; Uberuaga, B. P.; Jónsson, H. A climbing image nudged elastic band method for finding saddle points and minimum energy paths. *The Journal of Chemical Physics* **113**, (2000): 9901.

[13] Hermes, E. D.; Sargsyan, K.; Najm, H. N.; Zádor, J. Sella, an open-source automation-friendly molecular saddle point optimizer. *Journal of Chemical Theory and Computation* **18**, (2022): 6974.

[14] Li, J.; Zhan, G.; Yang, J.; Quan, F.; Mao, C.; Liu, Y.; Wang, B.; Lei, F.; Li, L.; Chan, A. W. Efficient ammonia electrosynthesis from nitrate on strained ruthenium nanoclusters. *Journal of the American Chemical Society* **142**, (2020): 7036.

[15] Wu, Z.-Y.; Karamad, M.; Yong, X.; Huang, Q.; Cullen, D. A.; Zhu, P.; Xia, C.; Xiao, Q.; Shakouri, M.; Chen, F.-Y. Electrochemical ammonia synthesis via nitrate reduction on Fe single atom catalyst. *Nature Communications* **12**, (2021): 2870.

[16] Liu, Y.; Zhuang, Z.; Liu, Y.; Liu, N.; Li, Y.; Cheng, Y.; Yu, J.; Yu, R.; Wang, D.; Li, H. Shear‐strained Pd single‐atom electrocatalysts for nitrate reduction to ammonia. *Angewandte Chemie International Edition* **63**, (2024): e202411396.

[17] Zhang, S.; Wu, J.; Zheng, M.; Jin, X.; Shen, Z.; Li, Z.; Wang, Y.; Wang, Q.; Wang, X.; Wei, H. Fe/Cu diatomic catalysts for electrochemical nitrate reduction to ammonia. *Nature Communications* **14**, (2023): 3634.

[18] Cheng, X. F.; He, J. H.; Ji, H. Q.; Zhang, H. Y.; Cao, Q.; Sun, W. J.; Yan, C. L.; Lu, J. M. Coordination symmetry breaking of single‐atom catalysts for robust and efficient nitrate electroreduction to ammonia. *Advanced Materials* **34**, (2022): 2205767.

[19] Yang, J.; Qi, H.; Li, A.; Liu, X.; Yang, X.; Zhang, S.; Zhao, Q.; Jiang, Q.; Su, Y.; Zhang, L. Potential-driven restructuring of Cu single atoms to nanoparticles for boosting the electrochemical reduction of nitrate to ammonia. *Journal of the American Chemical Society* **144**, (2022): 12062-12071.

[20] Guo, H.; Guo, Z.; Xue, G.; Wang, H.; Gong, J.; Chu, K.; Qin, J.; Guan, Y.; Dong, H.; Chen, Y. Entropy‐Driven Stabilization of Noble Metal Single Atoms: Advancing Ammonia Synthesis and Energy Output in Zinc‐Nitrate Batteries. *Advanced Materials* **37**, (2025): 2500224.

[21] Liu, K.; Sun, Z.; Peng, X.; Liu, X.; Zhang, X.; Zhou, B.; Yu, K.; Chen, Z.; Zhou, Q.; Zhang, F. Tailoring asymmetric RuCu dual-atom electrocatalyst toward ammonia synthesis from nitrate. *Nature Communications* **16**, (2025): 2167.

[22] Shen, F.; He, S.; Tang, X.; Liu, Y.; Wang, Y.; Yin, Y.; Lv, X.; Fu, W.; Zou, Y.; Jiang, G. Breaking Linear Scaling Relation Limitations on a Dual‐Driven Single‐Atom Copper‐Tungsten Oxide Catalyst for Ammonia Synthesis. *Angewandte Chemie International Edition* **64**, (2025): e202423154.

[23] Zhou, Y.; Zhang, L.; Wang, M.; Shen, X.; Zhu, Z.; Qian, T.; Yan, C.; Lu, J. Maximized atom utilization in a high-entropy metallene via single atom alloying for boosted nitrate electroreduction to ammonia. *Nature Communications* **16**, (2025): 7915.
